# Non-invasive parameters of autonomic function using beat-to-beat cardiovascular variations and arterial stiffness in hypertensive individuals: a systematic review

**DOI:** 10.1186/s12938-024-01202-6

**Published:** 2024-02-20

**Authors:** Jia Hui Ooi, Renly Lim, Hansun Seng, Maw Pin Tan, Choon Hian Goh, Nigel H. Lovell, Ahmadreza Argha, Hooi Chin Beh, Nor Ashikin Md Sari, Einly Lim

**Affiliations:** 1 Department of Biomedical Engineering, Faculty of Engineering, Universiti Malaya, 50603 Kuala Lumpur, Malaysia; 2 Quality Use of Medicines and Pharmacy Research Centre, UniSA Clinical and Health Sciences, University of South Australia, Adelaide, 5000 Australia; 3 Department of Mechatronics and BioMedical Engineering, Lee Kong Chian Faculty of Engineering and Science, Universiti Tunku Abdul Rahman, Bandar Sungai Long, Kajang, 43200 Selangor, Malaysia; 4 Ageing and Age‑Associated Disorders Research Group, Department of Medicine, Faculty of Medicine, Universiti Malaya, 50603 Kuala Lumpur, Malaysia; 5 Graduate School of Biomedical Engineering, UNSW Sydney, Sydney, NSW Australia; 6 Department of Primary Care Medicine, Faculty of Medicine, Universiti Malaya, Kuala Lumpur, Malaysia; 7 Division of Cardiology, Department of Medicine, Universiti Malaya, Kuala Lumpur, Malaysia; 8 Tyree Institute of Health Engineering (IHealthE), UNSW Sydney, Sydney, NSW Australia; 9 South West Sydney (SWS), School of Clinical Medicine, UNSW Sydney, Sydney, NSW Australia; 10 Woolcock Vietnam Research Group, Woolcock Institute of Medical Research, Sydney, Australia

## Abstract

**Purpose:**

Non-invasive, beat-to-beat variations in physiological indices provide an opportunity for more accessible assessment of autonomic dysfunction. The potential association between the changes in these parameters and arterial stiffness in hypertension remains poorly understood. This systematic review aims to investigate the association between non-invasive indicators of autonomic function based on beat-to-beat cardiovascular signals with arterial stiffness in individuals with hypertension.

**Methods:**

Four electronic databases were searched from inception to June 2022. Studies that investigated non-invasive parameters of arterial stiffness and autonomic function using beat-to-beat cardiovascular signals over a period of > 5min were included. Study quality was assessed using the STROBE criteria. Two authors screened the titles, abstracts, and full texts independently.

**Results:**

Nineteen studies met the inclusion criteria. A comprehensive overview of experimental design for assessing autonomic function in terms of baroreflex sensitivity and beat-to-beat cardiovascular variabilities, as well as arterial stiffness, was presented. Alterations in non-invasive indicators of autonomic function, which included baroreflex sensitivity, beat-to-beat cardiovascular variabilities and hemodynamic changes in response to autonomic challenges, as well as arterial stiffness, were identified in individuals with hypertension. A mixed result was found in terms of the association between non-invasive quantitative autonomic indices and arterial stiffness in hypertensive individuals. Nine out of 12 studies which quantified baroreflex sensitivity revealed a significant association with arterial stiffness parameters. Three studies estimated beat-to-beat heart rate variability and only one study reported a significant relationship with arterial stiffness indices. Three out of five studies which studied beat-to-beat blood pressure variability showed a significant association with arterial structural changes. One study revealed that hemodynamic changes in response to autonomic challenges were significantly correlated with arterial stiffness parameters.

**Conclusions:**

The current review demonstrated alteration in autonomic function, which encompasses both the sympathetic and parasympathetic modulation of sinus node function and vasomotor tone (derived from beat-to-beat cardiovascular signals) in hypertension, and a significant association between some of these parameters with arterial stiffness. By employing non-invasive measurements to monitor changes in autonomic function and arterial remodeling in individuals with hypertension, we would be able to enhance our ability to identify individuals at high risk of cardiovascular disease. Understanding the intricate relationships among these cardiovascular variability measures and arterial stiffness could contribute toward better individualized treatment for hypertension in the future.

*Systematic review registration*: PROSPERO ID: CRD42022336703. Date of registration: 12/06/2022.

## Introduction

Hypertension, a condition associated with an increased cardiovascular morbidity and mortality, represents a major global health issue [1]. Hypertension is prevalent in older people [2] and often relates to abnormal autonomic nervous system (ANS) function, with an observed overactivation of the sympathetic nervous system (SNS) [3–5]. Earlier studies have reported that elevated sympathetic outflow is associated with the development and progression of arterial fibrosis and stiffening [6, 7], a primary determinant of outcomes in the hypertensive population [8, 9]. In these studies, sympathetic nerve activity was assessed invasively by inserting tungsten microelectrodes into nerves projecting to the target muscles [10–12], while arterial stiffness was assessed by measuring aortic pulse wave velocity using invasive pressure catheters [13]. Due to their invasive nature, these measurements are not widely used or routinely performed in the clinic, thus limiting their prognostic value.

Blood pressure lowering medications aim to restore the ANS function and protect against target organ damage which occurs with untreated hypertension. Non-invasive and reliable assessment of both ANS function and arterial stiffness are required to characterize the effects of different blood pressure lowering medications on ANS function, and whether the observed effects then translate into improvements in arterial properties. Arterial stiffening, a well-established consequence of uncontrolled hypertension, is a recognized precursor to end organ damage. The challenges associated with determining both arterial stiffness and ANS, however, have led to a lack of understanding regarding the relationship between them. Non-invasive modalities for arterial stiffness assessment, which includes arterial tonometry, Doppler ultrasonography and magnetic resonance imaging, have now emerged [13–15]. Cardiovascular autonomic measurements, such as heart rate and blood pressure variabilities, have also received increased attention as a means of non-invasive ANS function assessment [16, 17]. Emphasis has grown about the significance of blood pressure variability (BPV) over traditional blood pressure measurements in hypertension [18–20]. While prior research utilizing 24-h ABPM or clinical BPV derived from multiple home visits have found associations with vascular alterations [21–26], these assessments often rely on visit-to-visit or 24-h blood pressure and heart rate measurements [23, 27]. These measurements are strongly influenced by the circadian rhythm and are dependent on patient cooperation, thus reducing the credibility of the derived autonomic indices [28]. Meanwhile, the heterogeneity of study populations and the limitations of intermittent blood pressure monitoring [29], may have raised questions about the consistency of the associations observed [21, 30].

Non-invasive, continuous beat-to-beat physiological recordings are acquired over a shorter period of time and have the potential of providing more reliable and reproducible alternatives for ANS functional assessment [31]. However, limited studies have explored the relationship between beat-to-beat BPV and arterial stiffness, despite its potential prognostic significance [20, 21, 32]. Continuous beat-to-beat BPV monitoring allows for the examination of rapid fluctuations in blood pressure, providing a more detailed and immediate understanding of autonomic control and its impact on vascular function. This can lead to earlier detection of hypertensive changes, ultimately enabling more timely interventions and personalized treatment strategies. By delving into the intricate interplay between these two factors, we can uncover vital insights into the pathophysiological mechanisms underlying hypertension and its associated complications.

To the best of our knowledge, no comprehensive review has systematically evaluated the correlation between various baroreflex sensitivity (BRS) indices, beat-to-beat cardiovascular variabilities (heart rate variability (HRV) and BPV) and arterial stiffness in hypertension. Filling this void holds the promise of improving risk prediction, refining management strategies, and ultimately advancing our ability to combat hypertension effectively. This endeavor is not just about connecting the dots, it is about illuminating the path toward a more nuanced and precise approach to hypertension care. In this review, autonomic nervous system measures encompass parameters, such as BPV and HRV (also referred to as beat-to-beat cardiovascular variabilities), BRS as well as hemodynamic changes in response to autonomic challenges. All these parameters were derived based on continuous, beat-to-beat measurement and variation of blood pressure and/or heart rate. These quantitative indices have been proven to be reproducible and comparable to the gold standard invasive measures [11]. This systematic review aims to (i) provide an overview of the experimental design and assessment techniques for ANS and arterial stiffness; and (ii) analyze the extent to which different quantitative indices of ANS function derived based on beat-to-beat cardiovascular variabilities are related to various non-invasive indicators for arterial stiffness, as well as exploring their bidirectional relationship. We suggested that apart from BRS parameters which require both heart rate and blood pressure measurements, beat-to-beat BPV parameters could serve as alternative, robust prognostic indicators for hypertension and are associated with non-invasive indicators of arterial stiffness. This study would shed light on the characterization of blood pressure regulatory pathways in hypertension using non-invasive, continuous measurements which are both reproducible and easily accessible at a lower cost [33].

## Results

### Study selection

**Fig. 1:**
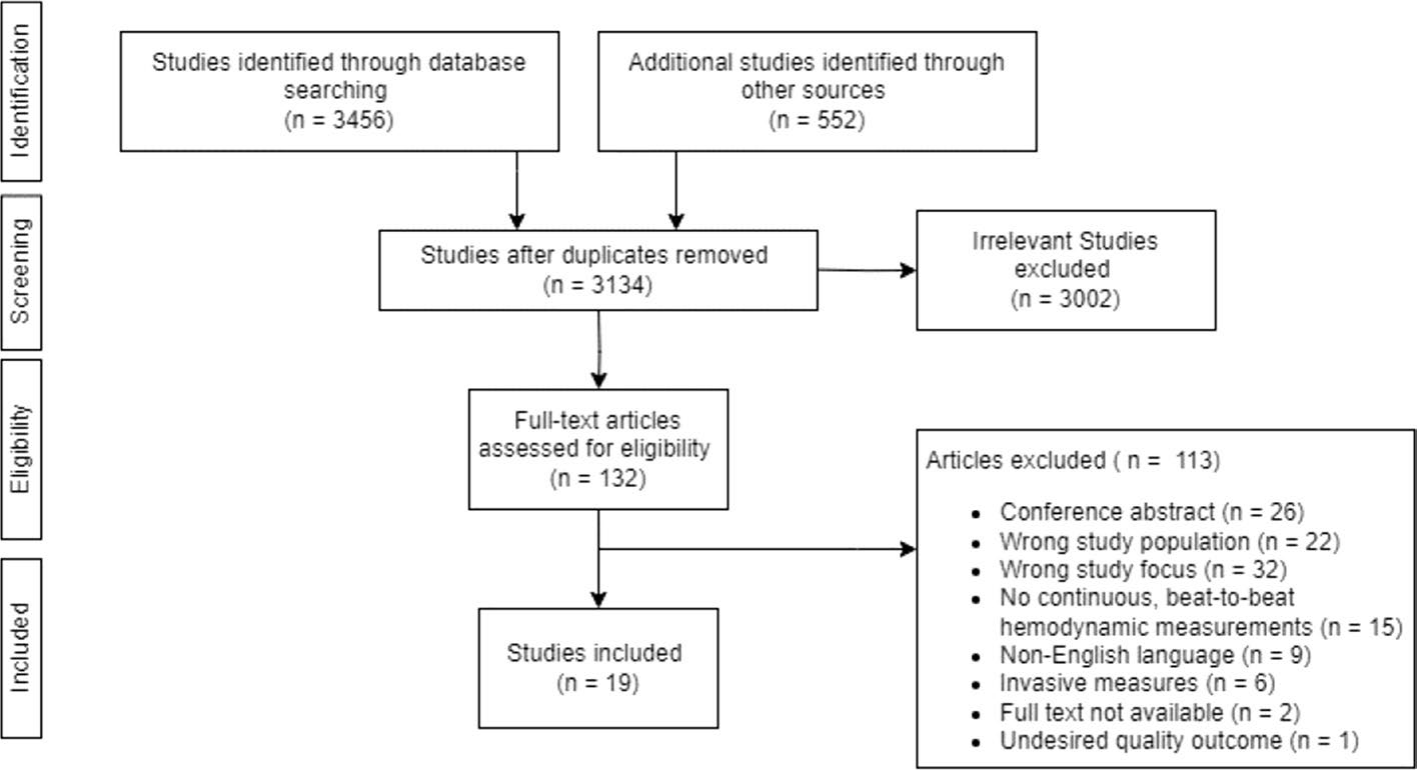
PRISMA (Preferred Reporting Items for Systematic Reviews and Meta-Analysis) study selection process

Figure 1 summarizes the process of study identification and selection. A total of 4008 studies were identified through the database search and other stated sources. After the removal of duplicates, 3134 studies were potentially eligible and were included for the abstract and title screening process. A total of 132 full-text studies were identified and evaluated for potential eligibility of which 19 studies met the inclusion criteria.

### Risk of bias

Table 1 summarizes the quality assessment results of all the 19 studies included in this review. None of the studies reported sample size calculations. Eight of the 16 checklist items were reported by all studies, two items were reported by 18 studies, one item by 16 studies, one by 13 studies and two items by 12 studies. Five studies fulfilled 15 of the 16 checklist items, eight studies fulfilled 14 items, three studies fulfilled 13 items, while the remaining studies fulfilled nine to 12 checklist items.

**Table 1:** Quality assessment for potential risk of bias

Authors (Year)	Checklist																Score
	1	2	3	4	5	6	7	8	9	10	11	12	13	14	15	16	
Cunha et al. [34]	/	/	/		/	/		/	/	/	/	/	/	/	/	/	14/16
Kosch et al. [35]	/	/	/		/	/				/	/	/	/	/	/		11/16
Lantelme et al. [36]	/	/	/	/	/	/		/	/	/	/	/	/	/	/	/	15/16
Tsai et al. [37]	/	/	/	/	/	/			/	/	/	/	/	/	/		13/16
Siegelova et al. [38]	/	/	/			/				/		/	/		/	/	9/16
Labrova et al. [39]	/	/	/	/	/	/			/	/	/	/	/	/	/	/	14/16
Novakova et al. [40]	/	/	/	/	/	/			/	/	/	/	/			/	12/16
Labrova et al. [41]	/	/	/	/	/	/		/	/	/	/	/	/		/	/	14/16
Chan et al. [ 42]	/	/	/	/	/	/			/	/	/	/	/	/	/	/	14/16
Honzikova et al. [43]	/	/	/		/	/			/	/	/	/	/	/	/	/	13/16
Michas et al. [44]	/	/	/	/	/	/		/	/	/	/	/	/	/	/		14/16
Celosvka et al. [45]	/	/	/		/	/		/	/	/	/	/	/	/	/	/	14/16
Tikkakoski et al. [46]	/	/	/	/	/	/		/	/	/	/	/	/	/	/		14/16
Okada et al. [47]	/	/	/	/	/	/		/	/	/	/	/	/	/	/	/	15/16
Manios et al. [48]	/	/	/	/	/	/		/	/	/	/	/	/	/	/		14/16
Xia et al. [20]	/	/	/	/	/	/		/	/	/	/	/	/	/	/		14/16
Celovska et al. [49]	/	/	/		/	/		/	/	/	/	/	/	/	/		13/16
Koletsos et al. [50]	/	/	/	/	/	/		/	/	/	/	/	/	/	/	/	15/16
Jiang et al. [51]	/	/	/	/	/	/		/	/	/	/	/	/	/	/	/	15/16
	19	19	19	13	18	19	0	12	17	19	18	19	19	16	19	12	
**Checklist**																	
Abstract	1. Describe a brief but informative and balanced summary of what has been done and found																
Introduction	2. Describe the related study background 3. Describe the specific objectives, including any potential hypotheses																
Methods	4. Describe the study protocol, including setting, locations, periods of recruitment or follow-up and data collection. (e.g., how the patients are recruited, where and when the recruitment was.) 5. Define the diagnostic criteria for disease as well as the distributions of outcomes, exposures, predictors, potential confounders, and effect modifiers in each subject group. (e.g., medication status, evidence of cardiovascular risk) 6. Explain the data sources and how they are measured 7. Explain the establishment of the study size with the confidence interval 8. Explain how the quantitative variables are handled in the analysis 9. Describe all the statistical methods used and/or the handling of the missing data																
Results	10. Describe the number of included participants in the study 11. Describe the characteristics of study participants, such as demographic, clinical or medication status 12. Clearly describe the main findings																
Discussion	13. Provide a summary of the key results with reference to study objectives 14. Discuss the study limitations, including the sources of potential bias 15. Interpret the overall results, including the objectives, limitations, multiplicity of analyses, results from other similar studies																
Other information	16. State the funding source or the role of funders for the study																

### Population characteristics

Table 2 summarizes the characteristics of selected studies. Two of the 19 studies are longitudinal studies [40, 42], 11 are case–control studies [20, 35, 37, 39, 43, 44, 46–48, 50, 51], while the remaining are cohort studies which only involve the hypertension group [34, 36, 38, 41, 45, 49]. 15 studies involved participants with a mean age ranging from 40 to 65 years [20, 34–37, 39–44, 46, 48–50], while three studies recruited older individuals aged above 65 years [45, 47, 51]. Of all included studies, 12 included both normotensive control and essential hypertensive subjects [43], with both mixed genders involved [20, 35, 37, 39, 40, 44, 46–48, 50, 51], whereas one study investigated the association between autonomic control and vascular condition in men with essential hypertension only [38]. Some involved only hypertensive subjects in their study [34, 36, 49].

**Table 2:** Summary of population characteristics and assessment tools

Author(s)	Years	Population		Criteria for hypertension	Measurement devices
		Healthy control	Experimental study group		
Cunha et al. [34]	1997	–	*n* = 80 (51 men); age: 49 ± 11 years; untreated, essential hypertension	SBP > 140 mm Hg and/or DBP > 90 mm Hg via sphygmomanometer measurements during 5 different consultations (in 2 months)	1. Finger plethysmograph: SBP, DBP, RRI 2. Transcutaneous doppler flow: cf-PWV
Kosch et al. [35]	1999	*n* = 15 (7 men); age: 42 ± 2 years	*n* = 15 (7 men); age: 45 ± 3 years; untreated, essential hypertension	DBP ≥ 90 mm Hg measured in sitting position on 3 different occasions	1. Pneumotrace: ECG and respiration 2. Doppler ultrasound: end diastolic and systolic diameter of the carotid and brachial artery
Lantelme et al. [36]	2002	–	*n* = 271 (148 men); age: 53.4 ± 12.5 years; untreated (*n* = 139) and treated (*n *= 132) hypertension	–	1. Finapres^®^: SBP, DBP 2. Standard bipolar ECG: RRI 3. Complior, and self-made device: cf-PWV
Tsai et al. [37]	2003	*n* = 19 (8 men); age: 40.5 ± 12.9 years	*n* = 23 (9 men); age: 44.4 ± 10.9 years; untreated, essential hypertension	SBP: 130–159 mmHg and/or DBP: 85–99 mmHg	1. Radial artery tonometry: SBP, DBP, MAP 2. Applanation tonometer: central aortic wave 3. Impedance cardiography: SV and TPR 4. ECG: HR
Siegelova et al. [38]	2004	–	*n* = 30 (all men); treated, essential hypertension	–	1. Finapres^®^: SBP, DBP, RRI 2. Doppler echocardiography: carotid IMT
Chan et al. [42]	2005	–	*n* = 10 (5 men); Age: 42 ± 4 years; treated hypertension with ESRD	–	1. Finapres^®^: SBP, DBP, RRI 2. B-mode ultrasonography: carotid IMT
Labrova et al. [39]	2005	*n* = 23 (7 men); age: 44.5 ± 8.1 years	*n* = 25 (11 men); age: 47.4 ± 9.2 years; treated, essential hypertension	SBP ≥ 140 mm Hg and/or DBP ≥ 90 mm Hg	1. Finapres^®^: SBP, DBP, RRI 2. B-mode ultrasonography: carotid IMT
Labrova et al. [41]	2005	*n* = 23 (7 men); age: 43.5 ± 8.1 years	n = 25 (11 men); age: 47.4 ± 9.2 years; treated, essential hypertension	SBP ≥ 140 mm Hg and/or DBP ≥ 90 mm Hg	1. Finapres^®^: SBP, DBP, RRI 2. B-mode ultrasonography: carotid IMT
Novakova et al. [40]	2005	*n* = 15 (6 men); age: 44 ± 9 years	*n* = 25 (10 men); age: 49 ± 10 years; treated, essential hypertension	SBP ≥ 140 mm Hg and/or DBP ≥ 90 mm Hg	1. Finapres^®^: SBP, DBP, RRI 2. B-mode ultrasonography: carotid IMT
Honzikova et al. [43]	2006	*n* = 23; age: 44.1 ± 8.1 years	*n* = 27; age: 47.2 ± 8.7 years; treated, essential hypertension	–	1. Finapres^®^: SBP, DBP, RRI 2. B-mode ultrasonography: carotid IMT
Michas et al. [44]	2012	*n* = 34 (35.3% men); age: 50 ± 12 years	*n* = 126 (49.2% men); age: 53 ± 9 years; untreated, essential hypertension	SBP ≥ 140 mm Hg and/or DBP ≥ 90 mm Hg	1. ECG: RRI 2. Finometer^®^: SBP, DBP 3. Complior: cf-PWV
Celovska et al. [45]	2012	–	*n* = 26 (15 men); treated hypertension with history of ischemic stroke age: 66 ± 10 years *n* = 30 (17 men); treated essential hypertension (without stroke) age: 65 ± 6 years	–	1. Collin^®^ CBM-700 monitor: SBP, DBP, RRI 2. Duplex ultrasonography: common carotid and carotid bulb IMT
Tikkakoski et al. [46]	2013	*n* = 232 (38% men); age: 42 ± 12 years	*n* = 155 (55% men); age: 49 ± 11 years; untreated, essential hypertension	supine laboratory BP ≥ 135/85 mmHg	1. Radial artery tonometer: SBP, DBP, MAP 2. SphygmoCor^®^ pulse wave analysis system: central aortic wave (aortic PP, AIx) 3. Whole body impedance cardiography device: HR, SV, CO, PWV
Okada et al. [47]	2013	*n* = 30 (15 men); age: 68 ± 1 year	*n* = 40 (20 men); age: 68 ± 1 year; untreated, essential hypertension ; *antihypertensive drugs stopped for 2 weeks prior for treated patients	awake 24 h ambulatory SBP: 135–159 and/or awake 24 h ambulatory DBP: 85–99 mmHg	1. Finger plethysmograph: SBP, DBP, MAP 2. ECG: HR 3. SphygmoCor^®^: cfPWV
Manios et al. [48]	2014	*n* = 40 (30% men); age: 54 ± 11 years	n = 45 (53% men); age: 54 ± 9 years; untreated, essential hypertension	24-h BP ≥ 130/80 mmHg	1. Finometer: SBP, DBP 2. Ultrasound: carotid IMT
Xia et al. [20]	2017	*n* = 80 (44 men); age: 49.5 ± 11.5 years	n = 81 (42 men); age: 56.7 ± 10.1 years; hypertension	SBP ≥ 140 mm Hg and/or DBP ≥ 90 mm Hg	Finometer: SBP, DBP, ECG, SV
Celovska et al. [49]	2017	–	*n* = 20 (10 men); high normal BP (untreated, prehypertension); age: 59 ± 8 years *n* = 20 (10 men) essential, treated hypertension age: 61 ± 13 years	high normal BP range: 130–139/85–89 mmHg; Hypertension: BP ≥ 140/90 mmHg	1. Collin^®^ CBM-700 monitor: SBP, DBP, RRI 2. Duplex ultrasonography: common carotid and carotid bulb IMT
Koletsos et al. [50]	2019	*n* = 28 (57.1% men); age: 43.8 ± 13.0 years	*n *= 31 (51.6% men); age: 47.6 ± 7.0 year; untreated, newly diagnosed essential hypertension *n* = 27 (59.3 men); age: 47.5 ± 11.6 years; masked hypertensives	Office BP ≥ 140/90 mmHg and daytime ABPM ≥ 135/85 mmHg	1. Finapres^®^: SBP, DBP, HR 2. Ultrasound: carotid IMT 3. SphygmoCor^®^: cf-PWV, AIx
Jiang et al. [51]	2022	*n* = 153 (79 men); age: 69.8 ± 8 years	*n* = 247 (142 men); age: 72.2 ± 8.2 years; treated hypertension	SBP ≥ 140 mm Hg and/or DBP ≥ 90 mm Hg	1. Finapres^®^ PRO: SBP, DBP 2. Omron^®^: brachial–ankle PWV (baPWV)

*AIx* augmentation index, *ba-PWV* brachial–ankle pulse wave velocity*, cf-PWV* carotid-femoral pulse wave velocity, *CO* cardiac output, *DBP* diastolic blood pressure, *ESRD* end-stage renal disease*, HR* heart rate, *IMT* intima–media thickness, *MAP* mean arterial pressure*, PP* pulse pressure, *RRI*R-R interval, *SBP* systolic blood pressure, *SV* stroke volume*, TPR* total peripheral resistance

Experimental study participants were either untreated individuals with hypertension who had never received any blood pressure lowering therapy [34–37, 44, 46, 48, 50] or individuals receiving treatment for hypertension [36, 38–40, 42, 43, 45, 49, 51]. Two studies required their participants with hypertension to stop their blood pressure lowering agents 2 weeks before the study [36, 47]. Classes of blood pressure lowering agents used by the participants included diuretics, angiotensin-converting enzyme (ACE) inhibitors, angiotensin-II receptor antagonists, calcium channel antagonists or ß-adrenoceptor antagonists. In addition, participants in all selected studies had no clinical evidence of hypertension-related complications, cardiovascular disease, stroke, diabetes mellitus or secondary cause of hypertension, except for two studies which included individuals with hypertension and ischemic stroke [45] and end-stage renal disease (ESRD) after receiving nocturnal hemodialysis [42].

### Experimental design and assessment techniques for ANS and arterial stiffness

To address the first aim of this review, the experimental protocol and assessment methods of each included study were reviewed and further broken down into ANS function assessment and arterial stiffness assessment techniques as shown in Tables 3, 4, 5 and 6.

**Table 3:** Methods used to derive BRS in the selected papers

Measures	Derivation of BRS
BRS sequence method [34, 36, 42, 45, 47, 49]	Identifying sequences of at least three consecutive beats, where both SBP and RRI either increase or decrease, and then calculating the average slope of the identified sequences within a defined time frame
BRS spectral method [38–41, 43, 45, 49]	Calculating the modulus or gain of the transfer function at a frequency of 0.1 Hz using the formula: $\mathrm{BRS }[{\text{ms}}/{\text{mmHg}}]= \frac{Gxy (f)}{Gxx (f)}$ Gxy(f): cross-spectral density between SBP and RRI; Gxx(f): power spectral density of SBP
BRSf [39, 40, 45]	Using the same formula as BRS spectral method, calculating the modulus at 0.1 Hz using the instantaneous values of the heart rate (in Hz) and SBP $\mathrm{BRS }[{\text{Hz}}/{\text{mmHg}}]= \frac{Gxy (f)}{Gxx (f)}$ Gxy(f): cross-spectral density between HR and RRI; Gxx(f): power spectral density of SBP
BRS alpha-index [36, 44]	Calculating the square root of the ratio of the spectral powers of RRI and SBP within a band of a particular frequency. In [36], alpha-index for LF band (0.04–0.15 Hz) was considered. In [44], both LF and HF band (0.20–0.35 Hz) were considered and combined alpha-index was calculated: 0.5 × [LF alpha-index + HF alpha-index]

*BRS* baroreflex sensitivity, *HF* high frequency, *LF* low frequency, *RRI* RR-interval, *SBP* systolic blood pressure

**Table 4:** HRV parameters used in the selected papers

HRV parameters	Definition	Physiological interpretation
Time-domain measure		
Standard deviation, SD (ms) [40, 41]	Standard deviation of RR-interval	Total HRV
Frequency-domain measure		
Low frequency power, LF (ms^2^) [35]	Spectral power in the low frequency band (0.04–0.15 Hz)	Cardiac sympathetic modulation
High frequency power, HF (ms^2^) [35]	Spectral power in the high frequency band (0.15–0.4 Hz)	Cardiac vagal modulation
Total power, TP (ms^2^) [35]	Total spectral power (0.01–0.5 Hz)	–
LF/HF ratio [35]	Ratio of LF power to HF power	Cardiac sympathovagal balance
Spectral power density at frequency of 0.1Hz (in absolute unit, ms^2^/Hz, and relative units) [40, 41]	–	Likely due to the baroreceptor reflex, which reflects the 0.1 Hz arterial blood pressure oscillations (Mayer wave) [52]

**Table 5:** BPV parameters used in the selected papers

BPV parameters	Definition	Physiological Interpretation
Time-domain measure		
Standard deviation, SD [20, 40, 41, 48]	Standard deviation of SBP or DBP $\sqrt{\frac{1}{n-1}\sum_{i=1}^{n}{({X}_{i}-\overline{X })}^{2}}$	Measures the absolute magnitude of overall variability of BP
Coefficient of variation, CV [51]	Dividing the SD by the average SBP or DBP level $\frac{SD}{\overline{X} }$	Relative measure of variability that normalized the standard deviation of BP against mean of BP
Residual standard deviation, RSD [20]	Square root of the total squared differences of data points from a linear regression of SBP or DBP values against time $\sqrt{\frac{1}{n-2}\sum_{i=1}^{n}{({X}_{i}-\widehat{{X}_{i}})}^{2}}$	Quantifies the extent of variability in blood pressure over time by excluding the impact of the possible drift in mean BP
Average real variability, ARV [20]	Average of absolute difference between adjacent SBP or DBP values $\frac{1}{n-2}\bullet \sum_{i=1}^{n}|{X}_{i+1}-{X}_{i}|$	Quantifies the BP measurements over time by considering the sequence of measurements
Variation independent of mean, VIM [20]	Proportional to SD/mean^*x*^, with *x* derived from curve fitting $k\bullet SD/{\overline{X} }^{m}$	Quantifies BP fluctuations that occur independently of mean BP level
Time Rate, TR [48]	First derivative of SBP or DBP values against time	Quantifies the degree and rate of BP fluctuation, often used to assess the speed or dynamics of BP fluctuations
Frequency-domain measure		
Spectral power density at frequency of 0.1Hz (in absolute, mmHg^2^/Hz and relative units) [40, 41]	–	Reflects the 10 s oscillation related to BP and vasomotor tone regulation, which refers to Mayer wave
Non-linear measure		
Multiscale entropy [51]	Entropy or recurrence in physiologic series over different temporal or spatial scales	Captures the irregularity of BPV fluctuations across multiple time scales

*DBP* diastolic blood pressure, *n* total number of BP values, *SBP* systolic blood pressure; ${X}_{i}$: set of BP measurement values; $\widehat{{X}_{i}}$: fitted values form linear regression of blood pressure values against time; *k* and *m*: obtained from a fitting curve of the form *y* = kx^p^ through a plot of SD of BP against mean BP

**Table 6:** Arterial stiffness parameters used in the selected papers

Parameters	Description	Vascular characteristic assessed
cf-PWV or PWV [34, 36, 44, 46, 47, 50]	Measures the speed at which the pressure wave travels from the carotid artery to the femoral artery	Aortic stiffness (by quantifying wave propagation speed in the aorta)
ba-PWV [51]	Measures the speed at which the pressure wave travels from the brachial artery (arm) to the ankle	Peripheral arterial stiffness (by quantifying wave propagation speed in the peripheral arteries)
Carotid IMT [38–41, 43, 45, 48, 49]	Measures the thickness of the inner layers of the carotid artery wall	Thickness of the carotid artery wall
AIx or AIx adjusted for heart rate [37, 46]	Measures the effect of reflected waves on central blood pressure waveform	Aortic stiffness (by analyzing the effect of wave reflection on central blood pressure waveform)
TAC [20, 37, 42]	Measures the compliance of arteries and their ability to accommodate changes in blood volume, using the formula: SV/PP	Arterial compliance
DC [35]	Measures the changes in diameter or cross-sectional area of arteries in response to changes in blood pressure	Local arterial distensibility

*AIx* augmentation index, *ba-PWV* brachial–ankle PWV, *cf-PWV* PWV between carotid and femoral arteries, *DC* distensibility coefficient, *IMT* intima–media thickness, *LF* spectral power at low-frequency band, *PP* pulse pressure, *PWV* pulse wave velocity, *SV* stroke volume*, TAC* total arterial compliance

#### Autonomic nervous system (ANS) assessment

**Fig. 2:**
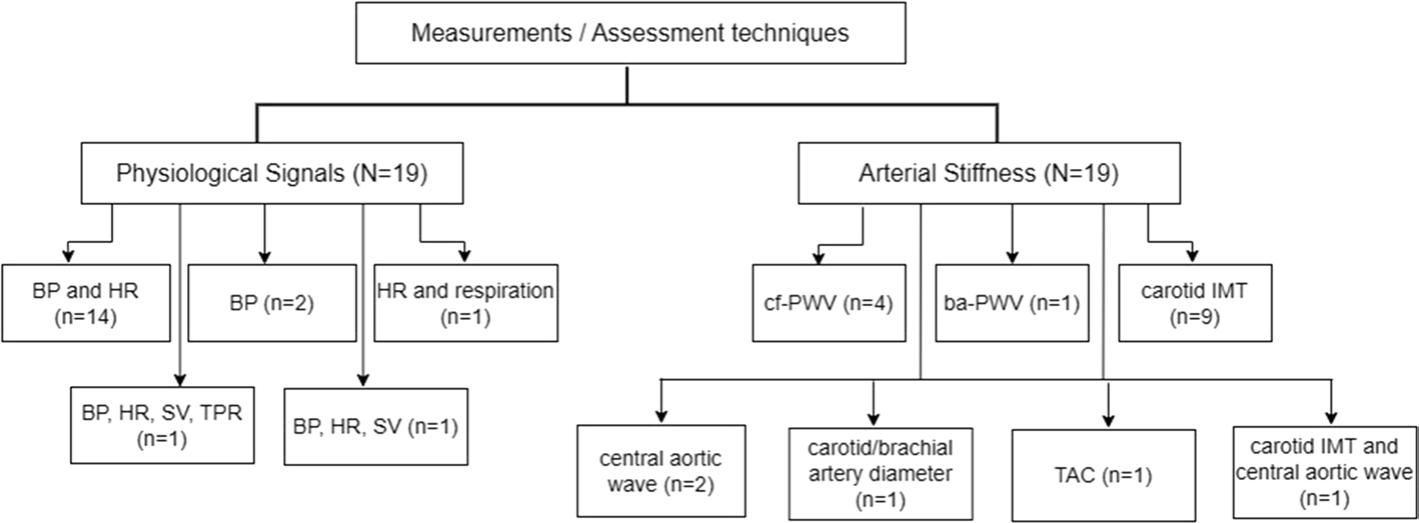
Physiological signal measurement and arterial stiffness assessment techniques used in the included studies. N = total number of included studies, n = number of studies. ba-PWV branchial–ankle pulse wave velocity, *BP* blood pressure, *cf-PWV* carotid-femoral pulse wave velocity, *HR* heart rate, *IMT* intima–media thickness, *SV* stroke volume, *TAC* total arterial compliance, *TPR* total peripheral resistance

**Fig. 3:**
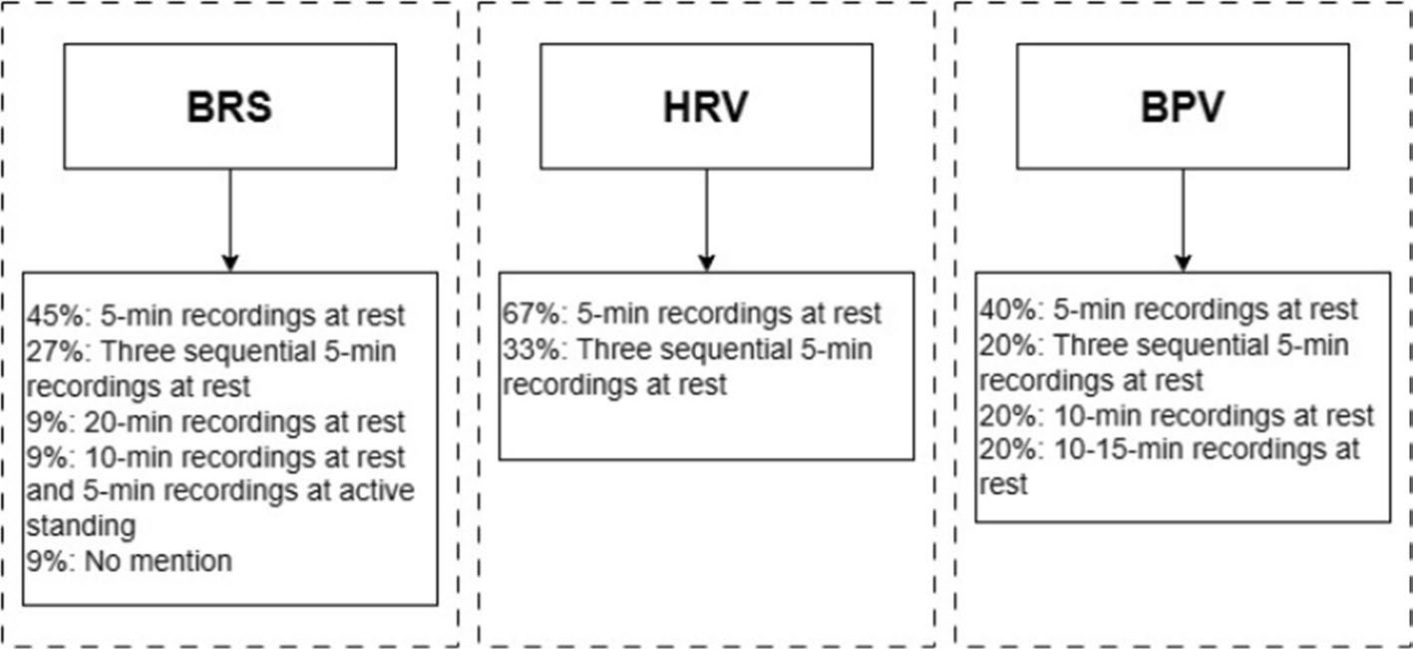
Duration of physiological signal recordings (blood pressure and RR interval) used in BRS, HRV and BPV analysis, with percentage of studies which analyzed the signal recordings. *BPV* blood pressure variability, *BRS* baroreflex sensitivity, *HRV* heart rate variability

Of the included studies, ANS function has been measured by BRS, BPV, HRV and hemodynamic changes in response to autonomic challenge tests. All of these assessments involved beat-to-beat recordings of physiological signals, as shown in Fig. 2. The experimental protocol and parameters involved are listed in Tables 3, 4 and 5. Figure 3 summarizes the duration of physiological recordings used in the quantitative ANS assessment.

##### Baroreflex sensitivity (BRS)

12 studies involved baroreflex sensitivity indices in quantifying the autonomic function, which involved beat-to-beat systolic blood pressure (SBP) and RR-interval (RRI) recordings. Of the 12 studies, BRS was generally quantified using the sequence method [34, 36, 42, 45, 47, 49] or spectral method [36, 38–41, 43–45, 49]. Specifically, spectral technique was used to estimate (i) the gain of transfer function between the changes in RRI or heart rate (HR) and changes in SBP at the frequency of 0.1 Hz; and (ii) alpha-coefficient. Other than the BRS mathematical derivation methods, BRS was assessed under different experiment protocols, such as spontaneous breathing [34, 36, 42], controlled breathing protocol using a metronome set at 0.33 Hz [38–41, 43, 45, 49] or 0.25 Hz [44], during standing [36] as well as after the end of Valsalva maneuver (Phase IV) [47]. Table 3 summarizes all the methods used by the studies which calculated BRS.

##### Heart rate variability (HRV)

Three studies involved short-term inter-beat interval (also known as heart rate variability) as a quantitative measure of autonomic function [35, 40, 41]. Two of the three studies measured SD and spectral power density at 0.1 Hz [40, 41], while the other one measured all the frequency-domain indices [i.e., low frequency (LF) power, high frequency (HF) power, total power (TP) and LF/HF ratio] [35].

Table 4 summarizes all the indices used in the studies which involved HRV.

##### Blood pressure variability (BPV)

Five studies measured very short-term BPV [20, 40, 41, 48, 51] based on supine finger blood pressure recordings.

Table 5 shows all the indices used in the studies which involved BPV, including systolic BPV (SBPV) and diastolic BPV (DBPV).

##### Autonomic challenge test

Five different types of autonomic challenge tests were used in the selected studies, which included mental stress test [37], handgrip test [50], head-up tilt (HUT) [46], Valsalva maneuver (VM) [47] and standing [36]. Three out of the five studies assessed changes in hemodynamics measurements from baseline, in response to a series of autonomic challenges [37, 46, 50].

#### Arterial stiffness assessment

Schematically, different assessment techniques were used for evaluating arterial stiffness non-invasively, as indicated in Fig. 2. These included the sonographic examination of carotid arteries [38–41, 43, 48–50], pulse wave velocity (PWV) [36, 44, 46, 47, 50], augmentation index (AIx) [37, 46, 50], total arterial compliance (TAC) [20, 37] and distensibility coefficient (DC) [35]. The details of measurement devices are listed in Tables 2. Table 6 shows all the parameters used to access the mechanical or structural properties of arteries in each study.

### ANS and arterial stiffness in hypertensive and normotensive subjects

The differences in ANS function and arterial stiffness between hypertensive and normotensive groups were examined as indicated in Tables 7, 8, 9 and 10. Hemodynamic parameters including blood pressure (BP), pulse pressure (PP), heart rate (HR), and total peripheral resistance (TPR) were significantly higher in the hypertensive group, both at baseline and in response to autonomic challenge tests. Hypertensive patients exhibited impaired baroreflex control with lower BRS, reduced HRV, and increased beat-to-beat BPV compared to the control group. In terms of arterial stiffness, the hypertensive group demonstrated significantly higher values of adjusted AIx, PWV, and carotid intima–media thickness (IMT), along with lower arterial compliance or distensibility, when compared to the normotensive subjects.

**Table 7:** BRS measures and arterial stiffness parameters

Author (s)	Years	Parameters involved		Experiment protocol		Association between BRS measure and arterial stiffness parameter	Other findings
		BRS measure	Arterial stiffness	BRS measure	Arterial stiffness		
Cunha et al. [34]	1997	BRS sequence method	cf-PWV	Continuous measurements of blood pressure for 20 min in the supine position to calculate beat-to-beat SBP, DBP and RRI; Spontaneous breathing	PWV between the carotid and femoral arteries was determined by the foot-to-foot method	Significant negative correlation between PWV and BRS in hypertensive subjects	–
Lantelme et al. [36]	2002	BRS sequence method (BRS-s) BRS alpha-index (BRS-a)	cf-PWV	Beat-to-beat measurements of SBP and RRI for 10 min in the supine position and 5 min in the standing position; Spontaneous breathing	PWV between the carotid and femoral arteries was determined by the foot-to-foot method	Significant negative correlation between both supine and standing BRS-s (not BRS-a) and PWV in hypertensive subjects	Significant increase in SBP, reduction in RRI, BRS-s and BRS-a from supine to standing
Siegelova et al. [38]	2004	BRS spectral method	Pulse pressure Carotid IMT	Continuous measurements of blood pressure for 5 min in the supine position; metronome-controlled breathing 0.33 Hz	B-mode ultrasonography was performed in the supine position on both the right and left common carotid artery Average carotid IMT was determined from 5 measurements	No specific analysis on the association between BRS measures and carotid IMT or pulse pressure was reported	Treated hypertensives with a higher pulse pressure are older, have a lower gain of the baroreflex and have a larger IMT
Labrova et al. [39]	2005	BRS spectral method BRSf	Carotid IMT	Continuous measurements of RR-intervals, beat-to-beat SBP and DBP in the sitting position at rest during a 5 min period; metronome-controlled breathing 0.33 Hz	B-mode ultrasonography was performed in the supine position on both the right and left common carotid artery Average carotid IMT was determined from 5 measurements	Significant negative correlation between carotid IMT and both BRS and BRSf in the whole group but not in hypertensive group	Decreased BRS and BRSf in hypertensive patients Positive correlation between age and IMT; negative correlation between age and BRS and BRSf in the whole group. However, age-related BRS was significantly weakened in the hypertensives due to an age-dependent prolongation of mean RRI
Novakova et al. [40]	2005	BRS spectral method BRSf	Carotid IMT	Continuous measurements of RR-intervals, beat-to-beat SBP and DBP in the sitting position at rest during a 5 min period; metronome-controlled breathing 0.33 Hz	B-mode ultrasonography was performed in the supine position on both the right and left common carotid artery Average carotid IMT was determined from 5 measurements	Significant negative association between BRS, BRSf and carotid IMT in the whole group (both measurements in a period of 1 year)	Greater IMT, decreased BRS and BRSf in hypertensive patients compared to normotensive subjects (both measurements in a period of 1 year, 2004–2005)
Labrova et al. [41]	2005	BRS spectral method BRSf	Carotid IMT	Continuous measurements of RR-intervals, beat-to-beat SBP and DBP in the sitting position at rest during a 5 min period; metronome-controlled breathing 0.33 Hz	B-mode ultrasonography was performed in the supine position on both the right and left common carotid artery Average carotid IMT was determined from 5 measurements	Significant negative correlation between carotid IMT and BRS and BRSf in the whole group but not in hypertensive group	Greater IMT, decreased BRS and BRSf in hypertensive patients compared to normotensive subjects
Chan et al. [42]	2005	BRS sequence method	TAC	RRI and SBP were derived from continuous ECG and non-invasive BP measurements, respectively, at two timepoints: (i) baseline: while receiving conventional hemodialysis ($\ge$ 18 h after the session); and (ii) 2 months after a stable dose of nocturnal hemodialysis ($\ge$ 4 h after the session) Spontaneous breathing	TAC (SV/PP)	Significant positive correlation between BRS and TAC in hypertensive subjects with ESRD	Following conversion from conventional hemodialysis to nocturnal hemodialysis, mean HR remains unaffected, SBP and DBP decreased, while BRS and TAC increased
Honzikova et al. [43]	2006	BRS spectral method	Carotid IMT	Continuous measurements of RR-intervals, beat-to-beat SBP and DBP in the sitting position at rest during a 5 min period; metronome-controlled breathing 0.33 Hz	B-mode ultrasonography was performed in the supine position on both the right and left common carotid artery Average carotid IMT was determined from 5 measurements	Significant negative correlation between carotid IMT and BRS in the normotensive and whole groups, but not in hypertensive group	BRS was negatively correlated with age and IMT in normotensive subjects and in the whole group, but not in hypertensive subjects
Michas et al. [44]	2012	BRS alpha-index	cf-PWV	Continuous measurements of RR-intervals, beat-to-beat SBP and DBP in the sitting position at rest during a 5 min period; metronome-controlled breathing 0.25 Hz	PWV between the carotid and femoral arteries was determined by the foot-to-foot method	No separate association analysis was reported between the two study groups Significant negative correlation between PWV and BRS in the whole group (normotensives and hypertensives)	Hypertensive patients were significantly older, had higher PWV and lower BRS than normotensive subjects
Celovska et al. [45]	2012	BRS sequence method BRS spectral method BRSf	Carotid IMT	Continuous measurements of RRI, SBP and DBP for three sequential periods of 5 min each in the supine position; metronome-controlled breathing 0.33 Hz	mean of the maxima at 4 sites of both common carotid artery and carotid bulb	Significant negative correlation between all BRS measures and carotid/carotid bulb IMT in hypertensive with stroke patients in comparison with stroke-free hypertensives	The higher the grade of hypertension, the lower the BRS/BRSf values Significant association between reduced BRS and increased SBP, PP and carotid IMT in hypertensives with stroke
Okada et al. [47]	2013	BRS sequence method (assessed by averaging the values of the slope of the linear correlation between RRI and beat-by-beat SBP during the two VM (phase IV))	cf-PWV	Continuous measurements of HR and BP during these tests: (i) 6 min of spontaneous breathing; (ii) two VM at 40 mmHg for 20 s with 5 min apart and (iii) 60° upright tilt for 10 min (MSNA data were collected during the last 3 min of tilting) Spontaneous breathing	PWV between the carotid and femoral arteries was determined by the foot-to-foot method	No specific analysis on the association between BRS measures and cf-PWV was reported	Higher cf-PWV and lower sympathetic BRS in hypertensive patients compared to normotensive subjects, but similar morning HR increase and cardiovagal BRS between both groups. Upright sympathetic BRS was smaller and %TPR/%MSNA by 60° HUT was higher in hypertensive subjects with greater morning surge than those with lesser morning surge. No difference in supine or upright MSNA between the groups. Significant positive correlation between morning surge and cf-PWV and sympathetic BRS in hypertensive subjects only. Sympathetic BRS, but not %TPR/%MSNA, was correlated with cf-PWV in hypertensive subjects.
Celovska et al. [49]	2017	BRS Sequence method; BRS Spectral method	Carotid IMT	Continuous measurements of RRI, SBP and DBP for three sequential periods of 5 min each in the supine position; metronome-controlled breathing 0.33 Hz	Measured on the far wall of common carotid artery along 1 cm long section proximal to the carotid bulb Mean of 3 single measurements at the side (left and right common carotid) with greater IMT was calculated	No specific analysis on the association between BRS measures and carotid IMT was reported	Significant negative correlation between spectral BRS, sequence BRS and systolic BP as well as mean BP in hypertensives Prehypertensive and hypertensives with critical BRS ≤ 5 ms/mmHg have significantly increased carotid and carotid bulb IMT

*BP* blood pressure*, BRS* baroreflex sensitivity, *cf-PWV* PWV between carotid and femoral arteries, *DBP* diastolic blood pressure, *HR* heart rate, *IMT* intima–media thickness, *MSNA* muscle sympathetic nervous activity, *PP* pulse pressure*, PWV* pulse wave velocity, *rel* relative unit*, RRI* RR-intervals, *SBP* systolic blood pressure*, TAC* total arterial compliance (SV/PP), *TPR* total peripheral resistance, *VM* Valsalva maneuver

**Table 8:** HRV measures and arterial stiffness parameters

Author (s)	Years	Parameters involved		Experiment Protocol		Association between HRV measure and arterial stiffness parameter	Other findings
		Quantitative HRV measure	Arterial stiffness	Quantitative HRV measure	Arterial stiffness		
Kosch et al. [35]	1999	LF, HF, LF/HF ratio, TP	Brachial and carotid artery DC	30 min recording of ECG and respiration in the supine position (8 am–10am) Spontaneous breathing	Vessel distensibility was measured by: 1. Relative systolic increase of vessel diameter: ratio between systolic increase of vessel diameter (Δd) and end diastolic diameter (d) (%); and 2. Arterial wall distensibility coefficient: (2 × Δd x d^−1^)/(SBP–DBP)	No separate association analysis was reported between the two study groups Significant negative correlation between carotid artery distensibility coefficient (DC) (not brachial artery) and LF/HF ratio in all subjects	Significant reduction in HRV (TP), carotid and brachial artery distensibility, as well as an increase in HRV (LF/HF) ratio, with a reduction in HF power (%) in hypertensive patients as compared to normotensive subjects
Novakova et al. [40]	2005	spectral power density at 0.1 Hz, SD	Carotid IMT	Continuous measurements of RRI, beat-to-beat SBP and DBP in the sitting position at rest during a 5 min period metronome-controlled breathing 0.33 Hz	B-mode ultrasonography was performed in the supine position on both the right and left common carotid artery Average carotid IMT was determined from 5 measurements	No specific analysis on the association between HRV measure and carotid IMT was reported	Greater IMT and decreased short-term variability in RR-intervals (absolute unit) at 0.1 Hz in hypertensive patients compared to normotensive subjects (both measurements in a period of 1 year, 2004–2005)
Labrova et al. [41]	2005	spectral power density at 0.1 Hz, SD	Carotid IMT	Continuous measurements of RRI, beat-to-beat SBP and DBP in the sitting position at rest during a 5 min period metronome-controlled breathing 0.33 Hz	B-mode ultrasonography was performed in the supine position on both the right and left common carotid artery Average carotid IMT was determined from 5 measurements	Significant negative correlation between carotid IMT and HRV SD in all subjects	Greater IMT, decreased short-term variability in RRI (SD and 0.1 Hz power) in hypertensive patients compared to normotensive subjects

*abs* absolute unit, *DBP* diastolic blood pressure, *DC* distensibility coefficient, *HF* spectral power at high-frequency band, *HR* heart rate, *HRV* heart rate variability, *IMT* intima–media thickness, *LF* spectral power at low-frequency band, *rel* relative unit*, RRI* RR-intervals, *SBP* systolic blood pressure*, SD* standard deviation*, TP* total power

**Table 9:** BPV measures and arterial stiffness parameters

Author (s)	Years	Parameters Involved		Experiment Protocol		Association between BPV measure and arterial stiffness parameter	Other findings
		Quantitative BPV measure	Arterial stiffness	Quantitative BPV measure	Arterial stiffness		
Novakova et al. [40]	2005	Spectral power density at 0.1Hz, SD	Carotid IMT	Continuous measurements of RRI, beat-to-beat SBP and DBP in the sitting position at rest during a 5 min period metronome-controlled breathing 0.33 Hz	B-mode ultrasonography was performed in the supine position on both the right and left common carotid artery Average carotid IMT was determined from 5 measurements	No significant association	Greater IMT and decreased DBP (relative unit) at 0.1 Hz in hypertensive patients compared to normotensive subjects (both measurements in a period of 1 year, 2004–2005)
Labrova et al. [41]	2005	Spectral power density at 0.1Hz, SD	Carotid IMT	Continuous measurements of RRI, beat-to-beat SBP and DBP in the sitting position at rest during a 5-min period metronome-controlled breathing 0.33 Hz	B-mode ultrasonography was performed in the supine position on both the right and left common carotid artery Average carotid IMT was determined from 5 measurements	No significant association	Greater IMT and decreased SBP (relative 0.1Hz power) and DBP (relative 0.1Hz power) in hypertensive patients compared to normotensive subject
Manios et al. [48]	2014	SD, TR	Carotid IMT	Continuous measurements of SBP and DBP for three sequential periods of 5 min each (10 am–12 pm) Spontaneous breathing	mean of the right and left IMT of the common carotid artery, calculated from 10 measurements on each side, taken 10 mm proximal to the carotid bifurcation	Significant positive correlation between carotid IMT and TR of beat-to-beat SBP variation in hypertensive patients	–
Xia et al. [20]	2017	SD, ARV, RSD, VIM	TAC	Continuous measurements of BP, ECG and SV in the supine position for 10 min Spontaneous breathing	SV/PP	SD, ARV, RSD, VIM of SBPV and DBPV were negatively correlated with TAC in hypertensive population Significant negative correlation between VIM of beat-to-beat SBP and TAC independent of SBP, DBP, age and BMI	Higher SBP, PP and SBPV, but reduced TAC and SV in hypertensive population as compared to the normotensive population. HR, DBP and DBPV indices were not significantly different between the 2 groups
Jiang et al. [51]	2022	CV, multiscale entropy	ba-PWV	Continuous measurements of SBP and DBP in the supine position for 10–15 min Spontaneous breathing	left- and right-side brachial–ankle pulse wave velocity	Significant negative correlation between BP complexity and ba-PWV in hypertensives	Within the hypertensive group, those with a longer duration of hypertension had significantly lower SBP and DBP complexity

*abs* absolute unit, *AIx* augmentation index, *ARV* average real variability, *ba-PWV* brachial–ankle pulse wave velocity, *BP* blood pressure, *cf-PWV* PWV between carotid and femoral arteries, *CV* coefficient of variation, *DBP* diastolic blood pressure, *DBPV* diastolic blood pressure variability*, DC* distensibility coefficient, *HR* heart rate, *IMT* intima–media thickness, *MAP* mean arterial pressure, *PP* pulse pressure*, PWV* pulse wave velocity, *rel* relative unit*, RRI* RR-intervals, *RSD* residual standard deviation, *SBP* systolic blood pressure, *SBPV* systolic blood pressure variability, *SD* standard deviation*, SV* stroke volume, *TAC* total arterial compliance (SV/PP), *TPR* total peripheral resistance, *TR* time-rate (first derivative of the BP values against time), *VIM* variation independent of mean

**Table 10:** Hemodynamic responses to autonomic challenges and arterial stiffness parameters

Author (s)	Years	Parameters Involved		Experiment Protocol		Association between hemodynamic responses and arterial stiffness parameter	Other findings
		Test	Arterial stiffness	Test	Arterial stiffness		
Tsai et al. [37]	2003	Mental stress test (SCWT)	AIx (corrected for heart rate at 75 bpm) TAC	Continuous BP and ECG measurements in a seated position, comprising 1. 6 min resting period (baseline) 2. 6 min mental stress test (SCWT) 3. 6 min recovery period	Three pairs of cardiac impedance (SV and TPR) and AIx (augmentation pressure/PP × 100%) measurements were taken and averaged during each of the three testing phases TAC (SV/PP)	No specific analysis on association between changes in hemodynamic measurements and AIx and TAC was reported In both normotensive and hypertensive groups, A concomitant increase in SBP, DBP, MAP, PP, HR and CO, with a decrease in TAC but not on AIx	Higher SBP, DBP, MAP, PP, HR, TPR, and adjusted AIx, but lower compliance in mildly hypertensive patients as compared to normotensive subjects
Tikkakoski et al. [46]	2013	HUT	Aortic pulse pressure, aortic reflection time and AIx (corrected for heart rate at 75 bpm) PWV	Continuous measurements of hemodynamic measurements for three consecutive 5 min periods: i. resting supine on the tilt table ii. HUT to 60 iii. tilt table was returned to the horizontal position The changes in response to HUT were calculated as differences in the mean values between the last three supine minutes preceding the head up tilt and the last 3 min during the head up tilt (when the signal was most stable)	Aortic pulse pressure, aortic reflection time and AIx were determined from the derived continuous aortic blood pressure waveform PWV was derived from the body electrical impedance changes obtained using the whole-body impedance cardiography device	No specific analysis on association between changes in hemodynamic measurements and aortic stiffness parameters was reported	In hypertensive patients with significantly higher supine PWV and SVRI, aortic SBP, and aortic PP decreased less, heart rate increased less, while aortic DBP and SVRI increased more during HUT, compared to normotensive individuals AIx was reduced during HUT in spite of a parallel increase in SVR but were not statistically significantly different between two groups after adjusted analysis
Koletsos et al. [50]	2019	Handgrip test	cf-PWV AIx (corrected for heart rate at 75 bpm)	Continuous measurements of BP and HR during (i) baseline in a seated position; (ii) handgrip test: three maximal isometric handgrip contractions with the dominant hand, with a 60 s rest between each measurement (maximal voluntary contraction, MVC = the highest of the three readings); (iii) 3 min submaximal handgrip exercise test (at 30% of MVC); and (iv) 3 min recovery SBP and DBP, and HR during baseline, handgrip exercise (averaged over the 3 min, and per minute assessments, that is, first, second, and third minute of exercise), and recovery were assessed. TPR was calculated and averaged per testing period	cf-PWV in supine position AIx (corrected for heart rate at 75 bpm) in supine position	BP rise during the first minute of isometric exercise was positively associated with resting PWV, while TPR response during exercise was positivity correlated with central/aortic SBP and DBP, AIx and PWV	Central/aortic BP, PWV and AIx were significantly higher in true hypertensive patients than normotensive individuals During exercise, individuals with true hypertensive exhibited a greater SBP/DBP response (increase) than normotensive individuals. HR did not differ significantly among groups in the respective testing periods No statistically significant difference in TPR among groups at baseline. During exercise, the true hypertensive patients showed significantly increased TPR

*AIx* augmentation index, *cf-PWV* PWV between carotid and femoral arteries, *CO* cardiac output, *DBP* diastolic blood pressure, *HR* heart rate, *HUT* head-up tilt, *IMT* intima–media thickness, *LF* spectral power at low-frequency band, *MAP* mean arterial pressure, *PP* pulse pressure*, PWV* pulse wave velocity, *RRI* RR-intervals, *SBP* systolic blood pressure*, SCWT* Stroop Color and Word Test, *SV* stroke volume, *SVR* systemic venous resistance*, SVRI* systemic venous resistance index*, TAC* total arterial compliance (SV/PP), *TPR* total peripheral resistance

### Association between quantitative ANS and arterial stiffness parameters

Result about the association between ANS and arterial stiffness in hypertensive subjects, measured using non-invasive techniques, were mixed. BRS indices, specifically those obtained via the spectral method during supine/sitting position, were the most commonly used method for quantifying the baroreflex control on the heart rate. On the other hand, sonographic examination of the carotid intima–media thickness (cIMT) was the most commonly used method for assessing the arterial stiffness.

#### Baroreflex sensitivity (BRS) measures and arterial stiffness parameters

12 studies involved BRS indices in quantifying ANS function, particularly the sympathetic and parasympathetic modulation of sinus node function. In terms of BRS sequence method, four out of five studies found a significant negative correlation with carotid IMT [45, 49] and cf-PWV (carotid–femoral pulse wave velocity) [34, 36]; positive correlation with TAC (SV/PP) [42] in the hypertensives. Seven quantified BRS using the spectral method and six of them revealed a significant negative relationship with carotid IMT [39–41, 43, 45, 49]. Four studies which quantified BRSf showed a significant negative correlation with carotid IMT [39–41, 45]. Two studies measured BRS alpha-index and cf-PWV [36, 44] but only one reported a significant association [44]. Whereas, no relevant association analysis between the BRS method and arterial stiffness parameters was reported in three studies [38, 47, 49], but a significantly lower BRS measure with a concomitant larger carotid IMT was identified in the hypertensive group [38, 49].

Table 7 summarizes the BRS measures and arterial stiffness parameters used together with their association.

#### Heart rate variability (HRV) measures and arterial stiffness parameters

Two of the three studies investigated the correlation between HRV indices (spectral power at 0.1Hz and standard deviation of RR-interval) and cIMT [40, 41]. Only one reported a significant negative correlation with cIMT in all subjects (control and hypertensive group) [41]. Another study which involved the frequency domain parameters of HRV (LF, HF, TP and LF/HF ratio) and correlated them with brachial and carotid artery distensibility coefficient, showed that only LF/HF ratio was negatively associated with carotid artery distensibility coefficient in hypertensive subjects [35]. Table 8 summarizes the HRV measures and arterial stiffness parameters used together with their association.

#### Very short-term blood pressure variability (BPV) measures and arterial stiffness parameters

In terms of BPV indices, two studies revealed a negative association between some of the SBPV/DBPV indices [i.e., standard deviation (SD), average real variability (ARV), residual standard deviation (RSD), variation independent of mean (VIM), complexity] with either TAC or PWV [20, 51], while one study found a positive correlation between SBPV/DBPV indices with carotid IMT [48].

Table 9 summarizes the BPV measures and arterial stiffness parameters used together with their association.

#### Hemodynamic changes to autonomic challenges with arterial stiffness parameters

Hypertensive subjects showed significant differences in their hemodynamic responses during autonomic function tests, such as head-up tilt (HUT) and handgrip exercise when compared to normotensive subjects [46, 50]. However, two out of the three studies did not report a specific analysis regarding the association between the changes in hemodynamic responses and arterial properties during the mental stress test and HUT test [37, 46]. In a study involving mental stress test, both the normotensive and mild hypertensive groups exhibited a similar response pattern. This pattern included a significant simultaneous increase in BP, HR and cardiac output (CO), along with a noteworthy decrease TAC in response to the test [37]. However, there was no significant change in TPR during the stress test [37]. On the contrary, during HUT, the untreated hypertensive group exhibited an exaggerated increase in TPR and BP, along with a less pronounced rise in HR [46]. Meanwhile, in response to the handgrip test, no significant change in HR was observed, compared to the normotensive group [50].

Two studies also measured AIx during the mental stress test and HUT test, but no significant change was observed in the mild hypertensive group [37] and untreated, established hypertensive group [46], in comparison with individuals with normal BP. The only study that performed an analysis on hemodynamic changes during the handgrip test and cf-PWV found a positive correlation between BP changes during the first minute of the test and resting cf-PWV [50].

Table 10 summarizes the hemodynamic responses and arterial stiffness parameters used together with their association.

## Discussion

The major findings from this study can be summarized as follows: (i) HRV LF/HF ratio is a more sensitive parameter in relation to arterial stiffness compared to other time- and frequency-domain parameters for HRV; (ii) SBPV has a greater discriminative ability for differentiating hypertensives from normotensives compared to DBPV; (iii) Beat-to-beat BPV measures, particularly VIM, time-rate, and multiscale entropy, appears to be more sensitive in relation to the changes in arterial properties; (iv) TPR plays a predominant role in BP regulation during HUT and handgrip test in individuals with established hypertension.

In this review study, there is considerable diversity in the measures employed to assess sympathetic or parasympathetic modulation of vascular tone and/or heart rate and arterial stiffness. The most frequently utilized parameters for quantifying these aspects are BRS and carotid IMT. Baroreflex sensitivity, beat-to-beat variations in blood pressure and heart rate, as well as changes in hemodynamics to physiological perturbations were altered in individuals with hypertension compared to normotensive individuals. These alterations have been found to be associated with non-invasive measures of arterial stiffness at baseline condition, including PWV, TAC, carotid IMT, and AIx.

### Experimental design and assessment techniques

To optimize the management of individuals with hypertension, it is important to characterize blood pressure regulation and understand the pathway through which blood pressure regulation is associated with autonomic nervous system function and vascular stiffness. However, this is impeded by a lack of standardized methods for autonomic function and arterial stiffness assessment, which may explain discrepancies in results among different studies. Furthermore, the patient selection criteria, which include the study population (treated or untreated hypertensives, patients with comorbidities), age (older vs middle-aged population) and sample size, are inconsistent across the selected studies.

In terms of autonomic function assessment, only a few studies observed hemodynamics changes in response to a number of autonomic challenge tests, while the remaining studies relied on measurements obtained in a supine or sitting position with different recording durations. The duration of physiological signal measurement used in the ANS analysis varies among included studies, but 5 min hemodynamic recordings were most commonly used. In addition, some studies performed controlled breathing during continuous, beat-to-beat hemodynamic recordings, while others used spontaneous breathing. More importantly, different indices have been used to quantify various aspects of ANS function, particularly the sympathetic and vagal modulation of sinus node function and vascular tone, such as BRS, HRV and BPV. However, it is crucial to note that there is no single universal index that can serve as a “gold standard” for assessing the entire ANS function. Instead, the appropriate index should be chosen based on the specific aspect of autonomic nervous system function that aims to be studied.

With regard to arterial stiffness assessment, pulse wave velocity (PWV) has been generally accepted as the gold standard method for evaluating aortic stiffness, and its association with autonomic dysfunction is well-established [53, 54]. Despite being the gold standard, PWV also has its own limitations as it is sensitive to the timing of wave reflection and blood pressure magnitude. Thus, alternative surrogate arterial stiffness measures have been introduced, which include carotid IMT, AIx, distensibility and TAC. Past studies have revealed a significant association between carotid IMT and PWV, indicating its ability to reflect arterial wall stiffness [55, 56]. However, carotid IMT, as a surrogate marker, has limitations. It primarily reflects structural changes related to atherosclerosis and may not fully capture the functional aspects of the arterial stiffness. In addition, carotid IMT may be influenced by local factors and might not represent the overall stiffness of the entire arterial system. Similar limitations are observed with AIx, distensibility and TAC, which may not exclusively represent arterial stiffness. For example, AIx which is an aortic stiffness measure, is dependent on wave reflections, heart rate and blood pressure, making the interpretation of its results challenging [57–59]. Distensibility measure primarily reflects local compliance, while TAC measure reflects the compliance of the entire arterial tree. Both of these measures are easily affected by blood pressure magnitude as vessel properties are nonlinear [60]. Overall, the complexity of arterial stiffness assessment requires multiple surrogate markers to complement the weakness of other measures.

### Mechanisms of autonomic alterations in hypertension and complications

The origin of essential hypertension remains a puzzle, but extensive discussions have revolved around the involvement of key systems: the renin–angiotensin system, the autonomic nervous system (ANS), body fluid volume and the peripheral vasculature [61, 62]. The ANS, steering short-term blood pressure changes, plays a crucial role in maintaining normal blood pressure levels. The alterations in cardiac autonomic control, whether preceding or following the onset of essential hypertension, contribute significantly to both functional and structural changes of the cardiac and subsequent systemic circulation [63]. Hence, increased or excessive sympathetic activity in hypertension is associated with increased arterial stiffness and left ventricular hypertrophy and subsequent target organ damage [64, 65].

Previous studies have identified the potentiating effect of the sympathetic drive in hypertension [66, 67], which is often then associated with baroreflex hypofunction [68]. Notably, heightened sympathetic activation in early hypertension results from impaired vagal control of heart rate or reduced baroreflex modulation of heart rate. It can be clinically presented as a hyperkinetic circulation marked by elevated HR, CO and a marginal increase in BP [63, 69, 70]. As hypertension takes root, there is a hemodynamic shift from a state of high cardiac output to one characterized by high vascular resistance [70].

The narrative centers on the transformations in the responsiveness of various cardiovascular organs in established hypertension. Cardiac remodelling due to the increased afterload, reduces the cardiac compliance of venous filling, leading to the gradual decrease of cardiac output in hypertension. In addition, reduced responsiveness to β-adrenergic stimulation explains the decreased cardiac output in established hypertension [70]. Vascular hypertrophy, a result of pressure-induced remodelling, explains the transformation, where the vessel wall becomes thicker and encroaches even more on the lumen, resulting in a steeper increase of vascular resistance (TPR) during vasoconstriction [70, 71]. As hypertension advances, vascular hyperresponsiveness to vasoconstriction requires less sympathetic firing (down regulation of sympathetic tone) to maintain the elevated blood pressure. Concurrently, vascular remodelling, a key contributor to arterial stiffness, impacts baroreceptor functionality. Stiffer vessel walls, common in hypertension-induced remodelling, limit the stretch and transmission of pressure changes to baroreceptors, attenuating their ability to normalize blood pressure and exert sympatho-inhibitory roles. Eventually the blunted baroreflex response leads to the reduced BRS and potentially reduced HRV, as well as greater BPV in hypertension [20, 34–36, 38–45, 47–49, 51]. The modifications in the baroreceptor–heart rate reflex (BRS) play a role in the reciprocal decrease of parasympathetic activity, leading to tachycardia and diminished HRV [72]. Simultaneously, the impairment of the baroreflex contributes to increased BPV, a phenomenon substantiated by earlier animal studies involving arterial baroreceptor denervation [73].

The apparent influence of the SNS on arterial stiffness does not definitively establish cause-and-effect relationships due to their mutual interdependence. The SNS, by inducing vasoconstriction, contributes to increased arterial stiffness. Conversely, arterial stiffness, in turn, influences the SNS through baroreceptor reflexes. The bidirectional impact of changes in aortic/arterial stiffness and SNS activity underscores the intricacy of their interactions. It is postulated that elevated aortic stiffness (i.e., cf-PWV, AIx) or arterial stiffness (i.e., carotid IMT and distensibility coefficient) parameters are associated with derangements in cardiovascular variability, characterized by reduced BRS and HRV, and greater BPV, stemming from the diminished sensitivity of baroreceptor in hypertension.

However, our findings reveal that various indices of BRS, HRV, and BPV were identified, but not all exhibited correlations with arterial structural changes. This discrepancy can be attributed to the inherent mathematical formulae governing these indices, as well as the impact of confounding factors such as respiration, age and the influence of antihypertensive medications. It is within the realm of speculation that the associations mentioned earlier in the context of hypertension might experience attenuation or even complete dissolution due to these contributing factors. Subsequent sections will meticulously explore the intricate relationships among diverse BRS, HRV, BPV indices, and parameters of aortic/arterial stiffness, contributing to a thorough understanding of these interconnected cardiovascular dynamics.

### Association between quantitative autonomic measures and arterial stiffness parameters

This review study confirms the well-established association between increased PWV and impaired autonomic control (evidenced by reduced BRS and HRV and increased BPV). Moreover, it also reveals similar negative associations between certain BRS, HRV and BPV measures and surrogate arterial stiffness indicators discussed in this review, including carotid IMT, AIx, and distensibility. An inverse association was identified between some BRS, HRV and BPV parameters and TAC, an arterial compliance measure which is inversely proportional to arterial stiffness.

#### Baroreflex sensitivity (BRS) measures

Baroreflex sensitivity (BRS) is a widely accepted, non-invasive method for assessing the baroreflex system's sensitivity. While the baroreflex primarily regulates blood pressure, BRS quantifies how effectively blood pressure returns to a setpoint after perturbation. However, assessing true BRS can be challenging experimentally, leading researchers to often use heart rate responses as a surrogate measure to gain insights into baroreflex system regulation. Available studies have demonstrated a lower supine or upright BRS (spectral or sequence method) in individuals with hypertension compared to normotensive individuals. The degree of impairment worsens with increasing severity (grade) of hypertension and in the presence of comorbidities, such as stroke and renal disease [42, 45].

In general, regardless of BRS derivation methods or arterial stiffness measures (i.e., either carotid IMT or cf-PWV), most papers revealed a significant negative association between BRS measure and arterial stiffness parameters. This implies that increased aortic or arterial stiffness is associated with diminished baroreflex function in individuals with hypertension. Arterial baroreceptors, which are specialized nerve endings located in the outer layers of the carotid sinus and aortic arch, respond to mechanical stretching of blood vessels [74]. Thus, the reduced compliance of aortic and carotid arteries due to the increased wall thickness or stiffness, very likely reduces the sensitivity of the baroreceptors in response to the blood pressure variations [38, 41, 44]. Our review findings indicate that studies using metronome-controlled breathing at specific frequencies, such as 0.33 Hz or 0.25 Hz, reported a more consistent correlation between frequency-domain BRS measures and arterial stiffness parameters [39–41, 43–45]. The use of metronome-paced breathing not only increases the BRS gain value [75], but also enhances the coherence and synchronization between respiratory and cardiovascular rhythms [76]. This approach allows for a more precise assessment of the relationship between BRS and arterial stiffness, which are both key indicators of cardiovascular health.

On the other hand, conflicting results emerged from two studies that examined BRS using the alpha-index concerning its association with aortic stiffness (cf-PWV) in hypertensive individuals [36, 44]. Apart from the difference in breathing protocol during the experiments, this discrepancy may also be attributed to the selection of frequency bandwidth during the derivation of BRS. Notably, the study reporting a significant association between BRS and cf-PWV estimated the alpha coefficient based on values obtained from both HF and LF band, while the study which did not find a significant correlation focused solely on the LF band. In general, HF components (0.15–0.4 Hz) reflect pressure oscillations associated with the respiratory mechanics, while LF components, including Mayer's waves (occurring every 10 s), are primarily linked to sympathetic activity [77, 78]. However, the origin of LF oscillation in heart rate remains debatable, with a potential involvement of vagal influences [79]. Incorporating both HF and LF components in the estimation of BRS offers a more comprehensive physiological perspective, as it reveals the interplay between sympathetic and vagal influences on heart rate regulation in response to blood pressure fluctuations [80].

Furthermore, the variation in the two study results may be influenced by antihypertensive treatments [36, 44]. The study demonstrating a significant association included untreated hypertensive participants [44], while the one without a significant correlation included a combination of treated and untreated hypertensive subjects [36]. Prior research indicates that long-term blood pressure control with angiotensin converting enzyme (ACE) inhibitors, calcium channel blockers (CCB) and beta-blockers may improve baroreflex function (increased BRS) and vascular function, but these improvements might not extend to changes in vascular structure [81–83].

In addition, some studies which conducted separate analysis in normotensive and hypertensive groups found that the association between BRS measures and carotid IMT was absent or weakened in the hypertensive group. Yet, this association became apparent when they considered the entire group [39–41, 43]. This suggests that other factors may contribute to the association beyond hypertension, such as the use of blood pressure lowering medication, which improved BRS through a reduction (increase) in sympathetic (vagal) activation [39, 43] as well as the aging factor [39, 41]. The dominance of blood pressure in hypertensive individuals is another critical factor [43]. Notably, the current blood pressure levels in individuals with hypertension may not accurately reflect the blood pressure conditions that initially contributed to the development of carotid IMT over time [39, 41, 43]. Thus, this underscores the significant influence of historical high blood pressure on carotid IMT in hypertensive individuals, potentially overshadowing the specific impact of BRS on IMT. Moreover, the lack of a significant association in hypertensive groups compared to the whole group analysis, may be attributed to small sample size [39–41, 43].

#### Heart rate variability (HRV) measures

The importance of HRV for evaluating the cardiac sympathovagal balance has been highlighted over decades [16]. Hypertensive patients have an altered cardiac sympathovagal balance (reflected by an increased LF/HF ratio), characterized by an increase in cardiac sympathetic activity, which is relative to reduced cardiac vagal modulation. The inverse relationship between the HRV LF/HF ratio and carotid artery distensibility [35] highlights that LF/HF ratio is likely to be a more sensitive parameter over other HRV frequency-domain indices (i.e., LF, HF power and TP), considering the relative changes in sympathetic and parasympathetic activities.

The mechanism responsible for the relationship between reduced HRV and increased arterial stiffness in hypertension remains unclear. However, hypertension leads to autonomic dysfunction, characterized by overactivation of the SNS [3–5]. This not only reduces HRV but also raises the resting heart rate [54, 84], which, in turn, contributes to arterial stiffness by altering blood flow dynamics and increasing shear stress [34]. In essence, HRV alone may not be directly related to arterial stiffness, but HRV parameters such as the LF/HF ratio potentially offer insights into how the autonomic nervous system influences the cardiovascular system, which can impact arterial stiffness. Unlike HRV LF/HF ratio, LF and HF components separately indicate specific aspects of autonomic activity and have opposing physiological interpretations. HRV HF power (0.15–0.4 Hz) reliably indicates cardiac vagal modulation and respiratory effects on heart rate, while the interpretation of HRV LF power (0.04–0.15 Hz) is debatable. Some viewed it as a marker of cardiac sympathetic activity [85], while others suggested that it reflects a combination of both sympathetic and vagal influences [16, 86], and some even suggested it primarily reflects parasympathetic activity [86].

Moreover, as observed in the same study [35], the correlation between altered sympathovagal balance and carotid artery distensibility, while not apparent in brachial artery distensibility, may be explained by reduced carotid artery distensibility. This reduction could lead to impaired carotid sinus sensitivity, potentially affecting the baroreceptor-mediated control of heart rate in hypertensive patients [87, 88].

In another study [41], carotid IMT was found to be significantly correlated with HRV SD, which is an established time-domain parameter in quantifying the HRV, but not spectral power density at 0.1 Hz [41]. This difference may arise from their distinct physiological interpretations. HRV SD encompasses both short-term high frequency variation (often parasympathetically mediated) and long-term low frequency components, and is strongly linked to frequency-domain parameters, such as LF, HF power and TP [16, 89]. In contrast, spectral power density at 0.1 Hz focuses on a specific 10 s oscillation associated with blood pressure and vasomotor tone regulation, which is potentially due to the sympathetic drive [86]. This might not fully capture the same comprehensive variation in heart rate as HRV SD does, explaining the insignificant correlation with carotid IMT.

#### Blood pressure variability (BPV) measures

In recent years, BPV has received increasing interest due to its association with target organ damage irrespective of mean blood pressure [21]. While most studies investigating the association between BPV and arterial remodeling have focused on visit-to-visit or short-term (i.e., 24 h) BPV, several studies have assessed beat-to-beat BPV as it is less susceptible to noise leading to better reproducibility [20, 40, 41, 48, 51]. In terms of blood pressure fluctuation, an increase in BPV has also been found to correlate with stiffening of the aorta or arteries, commonly occurring in hypertensive patients [20, 48, 51]. However, through our findings, only certain BPV parameters are associated with arterial structural or functional changes.

Similar to HRV, spectral power density at 0.1 Hz for SBPV or DBPV (in absolute and relative units) was not correlated with carotid IMT in the treated hypertensive subjects [40, 41], which can be elucidated by the effect of antihypertensive treatment [90]. In these individuals, there were no prominent signs of heightened sympathetic activity in the patients, and antihypertensive therapy effectively normalized their blood pressure. Importantly, the spectral power density of BPV at the 0.1 Hz frequency was notably suppressed [41]. To date, age-related changes in the structure of the arterial wall have been extensively studied [91], with numerous studies suggesting that these age-related alterations could potentially supersede correlations with BPV measures.

Based on our findings, SD of BPV has no association with arterial stiffness parameters. SD of BPV, which represents overall fluctuations around the mean blood pressure value, does not consider the chronological order of BP measurements and is susceptible to being affected by measurement errors that may arise during individual blood pressure readings. This limits its suitability for very short-term BPV calculations, especially when using non-invasive beat-to-beat digital BP measurements due to the significant noise associated with such measurements [20, 92]. Therefore, ARV, RSD and VIM were introduced to overcome the deficiencies of SD [93].

Time-domain indices which consider the time-series of the BP measurements such as ARV, RSD and VIM of BPV have shown promise in assessing autonomic function and were found to be correlated with total arterial compliance [20]. Specifically, ARV accounts for the time series of BP measurements, being less sensitive to low-frequency sampling of recordings; RSD excels in capturing the variability in BP fluctuations when a linear trend between BP fluctuations and time is present; and VIM, in its uniqueness, eliminates the influence of mean BP levels, showing the distinct contribution of these SBPV indices to TAC parameter in hypertensive individuals [20]. However, only VIM of SBP remained significantly associated with TAC, even after adjusting for age, body mass index (BMI), SBP and DBP, likely due to its ability to isolate the effect of mean BP levels, allowing it to detect subtle but clinically relevant variations in SBPV [93, 94]. Compared to DBPV indices, SBPV indices showed a stronger association with arterial stiffness parameter [20, 51]. Arterial stiffening restricts arterial wall stretch during systole, leading to an increase in the systolic aortic and pulse pressure, as well as greater fluctuations in systolic blood pressure [20, 27].

In addition to VIM of SBPV, TR of SBPV was shown to be correlated with carotid IMT, independent of SBP and DBP levels [20, 48]. Understanding the TR of BPV is crucial for grasping the impact of the speed and direction of blood pressure fluctuations on arterial stiffness [95, 96]. In fact, hypertensive groups have shown a similar positive link between the TR of 24 h ambulatory SBPV and carotid IMT [95]. This relationship suggests that swift changes in SBP can induce acute oscillatory shear stress on the vascular wall, ultimately leading to increased intima–media thickness and a consequent rise in arterial stiffness [95].

The non-linear measures of BPV, multiscale entropy shows its potential in revealing the blood pressure variations at different time scales or frequencies [51, 97]. BP regulation involves various elements, including cardiac output, vascular resistance, and neural and hormonal feedback mechanisms, all operating at different time scales. Traditional BP metrics based on single-scale fluctuation, such as mean level or variability, may not adequately characterize these intricate multiscale dynamics. Therefore, multiscale entropy provides a valuable tool to analyse and understand the complexity of BP regulation, offering insights into the underlying physiological processes and their interactions. It is shown that participants with a greater ba-PWV were associated with reduced multiscale entropy in SBP and DBP, implying that alterations in the vessel characteristics could disrupt blood pressure regulation (diminished blood pressure complexity) [51]. The specific association identified with multiscale entropy, as opposed to linear time-domain measure, such as CV [51], suggests that multiscale entropy is a more sensitive non-linear BPV parameters, allowing the capture of subtle changes within cardiovascular systems that might be overlooked by linear measures, and providing more insights into multiscale nature of blood pressure regulation.

Despite these studies, the causal relation between BPV and arterial stiffness measures remains unclear. It is uncertain if arterial stiffening leads to a reduction in baroreceptor sensitivity and thus increased BPV, or that conversely, enhanced blood pressure fluctuations lead to a deterioration in the elastin component of the arterial wall and thus arterial stiffening. A cyclical relationship between BPV and arterial stiffening may also exist.

#### Hemodynamic responses to autonomic challenges

In a study involving a mental stress test (SCWT), individuals with mild hypertension, despite having higher baseline BP, HR, and TPR, showed similar patterns of hemodynamic changes compared to those with normal BP [37]. During the test, both groups exhibited increased SBP and DBP due to heightened HR and subsequent increased CO. Notably, there were no significant changes in stroke volume (SV) and TPR in either group. This suggests that individuals with mild hypertension do not exhibit exaggerated blood pressure reactivity, and there were no notable differences in heart rate reactivity compared to normotensive participants. In contrast to established hypertension (characterized by reduced cardiac responsiveness and vascular hyperresponsiveness to stress) [98], mild hypertensives in this study did not exhibit vascular hyperresponsiveness (i.e., a significant increase in TPR) [37]. In summary, mental stress test primarily induces HR changes and not TPR changes, indicating that sympathetic nervous activity is not excessively stimulated in individuals with mild hypertension. The primary mechanism underlying the blood pressure response to mental stress in this group is likely the withdrawal of vagal tone [37].

Meanwhile, in response to SCWT, only a reduction in TAC but not adjusted AIx and TPR, was observed during the stress test [37]. TAC, reflecting overall vessel compliance, is easily affected by blood pressure magnitude due to nonlinear vessel properties [60]. Hence, the increase in blood pressure during SCWT led to a reduction in TAC, primarily resulting from changes in peripheral arterial compliance rather than properties of the central artery [37]. On the other hand, AIx, a measure of aortic distensibility, is predominantly determined by the distance the wave travels from the reflection site to the aorta [99]. Given the lack of significant change in TPR during the stress test, the location of wave reflection remained constant, and AIx remained unchanged [37].

Individuals with established hypertension, characterized by significantly higher aortic stiffness (measured through AIx and cf-PWV) and vascular resistance, exhibited distinct hemodynamic responses during HUT and handgrip test, compared to normotensive individuals [46, 50]. In contrast to mental stress test, both untreated hypertensive and normotensive groups showed an exaggerated increase in blood pressure and TPR during HUT and handgrip exercise tests, while their heart rate responses were similar [46, 50]. These findings indicate that the exaggerated BP increase was primarily driven by increased TPR, indicating greater sympathetic vascular activity through heightened adrenergic responsiveness in hypertensive individuals [47, 98]. Meanwhile, another study measuring sympathetic BRS during spontaneous breathing and 60° upright tilt, along with cardiovagal BRS during VM, revealed that elderly hypertensive individuals had comparable cardiovagal BRS but significantly smaller sympathetic BRS compared to normotensive subjects [47]. The observed smaller supine and upright sympathetic BRS, along with the pronounced TPR increase in hypertensive subjects during 60° upright tilt position, suggests that baroreflex control of TPR and increased vasoconstrictor sensitivity during orthostatic stress play a more predominant role that HR in the regulation of blood pressure in elderly hypertensive patients [47].

Furthermore, despite an increase in TPR during the HUT test, AIx (normalized to heart rate at 75 bpm) was reduced in both the normotensive and hypertensive groups [46]. This reduction was likely due to the reduced SV in response to the postural changes [46, 100]. In a study which performed the association analysis between hemodynamic changes during autonomic challenge and aortic stiffness, an increase in BP during the first minute of handgrip test was found correlated with the resting cf-PWV [50]. The author speculated that increased aortic stiffness might be the cause of abnormal rise in BP during the handgrip test [50]. However, further investigations are needed to determine the contributors to excessive BP response in hypertension and whether increased aortic stiffness causes exaggerated BP response or vice versa.

### Future directions and study limitations

To more accurately characterize autonomic regulation of cardiovascular function (sympathetic and vagal modulation of sinus node and vascular tone) and arterial properties as well as their association in hypertension, a standardized experimental design, suitable indices to quantify the ANS and arterial stiffness as well as additional hemodynamic measurements are required. Among the quantitative ANS indices, beat-to-beat BPV appear to be a promising option as recent studies have highlighted that enhanced fluctuation of blood pressure induced target organ damage, such as left ventricular hypertrophy, vascular stiffness and stroke. SBPV has a larger discriminative power in differentiating hypertensive from normotensive as compared to DBPV. As beat-to-beat BPV is not only influenced by autonomic dysfunction, there is a need to identify factors leading to excessive fluctuations in blood pressure before it can be utilized in routine clinical practice. Previous research studies only analysed BP and HR in assessing BP regulation, which is insufficient as BP is determined by both TPR and CO. With respect to arterial stiffness, TAC, a less well-established approach which reflects overall compliance of the entire arterial system, deserves greater attention when assessing the influence of autonomic dysfunction on arterial stiffness in patients with hypertension.

Based on our review, it is speculated that cardiovascular variability measures (BRS, HRV or BPV) correlate with arterial/aortic stiffness in hypertension. However, it is essential to note that the observed correlation does not establish definitive cause-and-effect relationships, given the mutual interdependence of these variables. With limited available literature, to what extent the above-mentioned association would be affected by hypertension remains unclear, as there are confounding factors which could contribute to the mentioned association, such as aging and the effect of antihypertensive drugs. Our review encountered challenges in drawing conclusive insights from the 19 relevant studies included, primarily due to the small sample sizes within those studies that compared the specific association between hypertensive and normotensive individuals [35, 37, 39–41, 43, 50]. Therefore, we have not included this comparison between the two populations in terms of the association in this current paper due to the insufficient evidence. It is noteworthy to highlight the limited scope of available research on this topic, underscoring the need for more comprehensive investigations in future studies.

A limitation of our work is that the included studies did not determine the effect of blood pressure lowering medications on sympathetic or parasympathetic modulation of vascular tone and/or heart rate and arterial properties. In addition, to establish the causal relationship between ANS function and arterial stiffness, interventions which potentially alter autonomic function or arterial stiffness, including lifestyle changes and certain medications, should be considered. In this systematic review, data or signal pre-processing techniques applied prior to the beat-to-beat cardiovascular variability analysis are not interpreted due to a lack of comprehensive description in most of the included studies. The findings of a correlation between non-invasive quantitative measures of autonomic function and arterial stiffness in essential hypertension, however, highlight the potential for non-invasive beat-to-beat blood pressure and heart rate measurements in providing individualized, targeted treatment for hypertension. Given the limited number of studies investigating beat-to-beat BPV measures in relation to arterial stiffness parameters, as well as the cross-sectional study design of existing research, there is a need for further investigation into the bidirectional relationship between beat-to-beat BPV and arterial structural or mechanical changes. However, our findings suggest that beat-to-beat BPV measures, particularly VIM, time-rate, and multiscale entropy, show potential as more sensitive indicators for correlating with arterial stiffness and may possess greater prognostic significance compared to BRS and HRV. This could signify a move from traditional snapshot office or home measurements of blood pressure toward a more detailed characterization of blood pressure profiles using very short-term office-based beat-to-beat hemodynamic measurements, which had been limited prior due to the challenges of longer-term blood pressure measurements.

## Conclusion

Non-invasive, beat-to-beat physiological measurements have a potentially useful role in characterizing sympathetic and vagal modulation of vascular tone and heart rate, and its relationship with arterial stiffness in individuals with hypertension. In general, hypertension is significantly associated with impaired autonomic control, as represented by the quantitative ANS indicators (BRS, HRV, BPV and hemodynamic changes in response to autonomic challenges) based on non-invasive, continuous hemodynamic measurements. In addition, the non-invasively measured arterial properties were found to be altered in hypertension. Different BRS, HRV and BPV indices were identified but not all of them were correlated with the arterial structural changes. Although the interpretation of available studies was limited by heterogeneity, a significant correlation between certain ANS parameters and arterial stiffness in hypertensive subjects was identified in most studies. Beat-to-beat BPV parameters are potentially more sensitive in correlating with arterial stiffness, in particular SBPV has a larger discriminative power in differentiating hypertensive from normotensive as compared to DBPV. Future standardization of the ANS and arterial stiffness assessment is required to better characterize factors causing hypertension in individual patients, which could help in devising better treatment strategies for hypertension in a personalized manner using non-invasive, beat-to-beat physiological recordings.

## Methods

The systematic review was reported with reference to the Preferred Reporting Items for Systematic Reviews and Meta-analyses 2020 (PRISMA-S) checklist [101].

### Sources

Four major electronic databases, National Library of Medicine (PubMed), Web of Science, Embase via Ovid platform and Scopus, were searched from inception until June 2022. The reference lists of articles included were also examined.

### Search strategy

Four main key concepts were identified, namely, beat-to-beat, autonomic nervous system, arterial stiffness and hypertension. The relevant search terms (Table 11) were used in each database with no restriction applied on the language and type of articles. The full electronic search in PubMed is presented in Table 12 and a similar strategy was replicated in the other databases.

**Table 11:** Keywords

	Key concept	Search terms	MeSH terms
#1	Beat-to-beat	“Beat-to-beat” **OR** “very short term” **OR** “ultra short term” **OR** “finger blood pressure” **OR** photoplethysmography **OR** Finapres **OR** Finometer **OR** “Task Force Monitor” **OR** Continuous **OR** Noninvasive	–
#2	Autonomic nervous system	Sympathetic **OR** parasympathetic **OR** vagal **OR** autonomic **OR** baroreflex **OR** baroreceptor **OR** “Valsalva maneuver” **OR** “tilt table” **OR** “head-up tilt” **OR** “deep breathing” **OR** “cardiovascular variability” **OR** “heart rate variability” **OR “**heart rate fluctuations” **OR** “blood pressure variability” **OR** “blood pressure fluctuations” **OR** “heart rate changes” **OR** “blood pressure changes” **OR “**time domain” **OR “**frequency domain” **OR** “valsalva ratio” **OR** handgrip **OR** “isometric exercise” **OR** “cold pressor test” **OR** “active standing” **OR** “lower body negative pressure”	• Autonomic nervous system • Baroreflex • Sympathetic nervous system • Parasympathetic nervous system
#3	Vascular condition	“Arterial stiffness” **OR** “total arterial compliance” **OR “**pulse wave velocity” **OR** “pulse wave analysis” **OR** distensibility **OR** compliance **OR** elasticity **OR** “pulse transit time” **OR** “peripheral resistance” **OR** “vascular aging” **OR** “augmentation index” **OR** ultrasonography **OR** ultrasound **OR** tonometry **OR** MRI **OR** “intima-media thickness”	• Vascular Remodeling/physiology • Vascular capacitance • Vascular resistance • Vascular stiffness
#4	Hypertension	“High blood pressure” **OR** Hypertensi* **OR** “elevated blood pressure” **OR** “raised blood pressure” **OR** “increased blood pressure”	• Hypertension • High blood pressure

Combination search: #1 AND #2 AND #3 AND #4

**Table 12:** Full search strategy in PubMed database

((((''Autonomic Nervous System"[Mesh] OR "Baroreflex"[Mesh] OR "Tilt-Table Test"[Mesh] OR "Valsalva Maneuver"[Mesh] OR "Autonomic Nervous System"[All Fields] OR "Baroreflex"[All Fields] OR "Tilt-table Test"[All Fields] OR "Valsalva Maneuver"[All Fields] OR sympathetic[All Fields] OR parasympathetic[All Fields] OR vagal[All Fields] OR "autonomic dysfunction"[All Fields] OR "baroreceptor sensitivity"[All Fields] OR "baroreflex sensitivity"[All Fields] OR autonomic[All Fields] OR "Valsalva maneuver" [All Fields] OR "head-up tilt" [All Fields] OR "Cold pressor test*"[All Fields] OR "deep breathing" [All Fields] OR "active standing" [All Fields] OR "postur*"[All Fields] OR "lower body negative pressure" [All Fields] OR handgrip[All Fields] OR "isometric exercise"[All Fields] OR "cardiovascular variability"[All Fields] OR "heart rate variability" [All Fields] OR "valsalva ratio"[All Fields] OR "blood pressure variability"[All Fields] OR "heart rate fluctuation*"[All Fields] OR "blood pressure fluctuation*"[All Fields] OR spectral[All Fields] OR "time domain"[All Fields] OR "frequency domain"[All Fields]) AND ("Vascular Remodeling/physiology"[Mesh] OR "Vascular Capacitance"[Mesh] OR "Vascular Resistance"[Mesh] OR "Vascular Stiffness"[Mesh] OR "Pulse Wave Analysis"[Mesh] OR "arterial stiffness"[All Fields] OR "total arterial compliance"[All Fields] OR "pulse wave velocity"[All Fields] OR "pulse wave analysis"[All Fields] OR "distensibility"[All Fields] OR compliance[All Fields] OR "vascular elasticity"[All Fields] OR "pulse transit time"[All Fields] OR "peripheral resistance"[All Fields] OR "vascular condition*"[All Fields] OR "vascular aging*"[All Fields] OR MRI[All Fields] OR "tonomet*"[All Fields] OR "augmentation index"[All Fields] OR ultrasound[All Fields] OR ultrasonography[All Fields] OR "intima-media thickness"[All Fields])) AND ("hypertension"[MeSH Terms] OR hypertensi*[All Fields] OR "high blood pressure"[All Fields] OR "raised blood pressure"[All Fields] OR "elevated blood pressure"[All Fields] OR "increased blood pressure"[All Fields])) AND ("beat-to-beat"[All Fields] OR "very short term"[All Fields] OR "ultra short term"[All Fields] OR Finapres[All Fields] OR "Task Force Monitor"[All Fields] OR "short term"[All fields] OR "finger blood pressure"[All Fields] OR "finger arterial pressure"[All Fields] OR continuous[All Fields] OR "non-invasive"[All Fields])) NOT (animal OR rat)	

### Inclusion and exclusion criteria

This review included only studies using non-invasive, continuous (beat-to-beat) hemodynamic measurements and excludes those involving long-term changes in blood pressure and heart rate. Inclusion criteria were studies that investigated: (1) the association between autonomic function and arterial properties using only non-invasive measurements; (2) primary or secondary hypertension with the resting systolic blood pressure (SBP) ≥ 140 mmHg and/or diastolic blood pressure (DBP) ≥ 90 mmHg (or ≥ 135/85 mmHg for home blood pressure measurements); (3) adults aged 18 years and above; (4) non-invasive assessment of the arterial properties; and (5) non-invasive assessment of the autonomic function, as derived from continuous, non-invasive, beat-to-beat physiological measurements over a period of ≥ 5 min: beat-to-beat blood pressure variability (BPV) and/or heart rate variability (HRV) in time or frequency domain, or baroreceptor sensitivity, or indices based on hemodynamic changes in response to autonomic function tests.

We excluded human studies which did not make autonomic control and arterial properties their main focus, and all animal studies. Studies that focused on whitecoat, masked, borderline, preeclampsia or gestational, or orthostatic hypotension were also excluded.

### Data extraction

All papers retrieved from the electronic database search process were imported into a reference management software (EndNote Version X10, Clarivate Analytics), followed by the removal of duplicates. These processes were performed independently by two authors (OJH, SH) and any disagreements were resolved by a third author (EL). Data of each included study were extracted by OJH and validated by SH using a data extraction form (Microsoft Excel 2021). The extracted data were reported narratively rather than quantitatively due to the variations in study designs and outcome measures.

### Data outcome

Non-invasive ANS measures include BRS, HRV and BPV, as well as hemodynamic changes in response to autonomic challenge tests. All measures were derived from non-invasive, continuous and beat-to-beat physiological signals.

#### Baroreflex sensitivity (BRS)

Baroreflex sensitivity, derived based on the spontaneous fluctuations in systolic arterial pressure and the RR intervals, is an established assessment tool for cardiac autonomic control [102]. Although various techniques for spontaneous BRS estimation have been introduced, only spectral analysis and the sequence methods were used in the selected studies [103, 104].

#### Beat-to-beat blood pressure variability (BPV) and heart rate variability (HRV)

Very short-term BPV refers to beat-to-beat variation in blood pressure over seconds to minutes [105]. The changes in time intervals between adjacent heartbeats are defined as HRV [16]. Both very short-term BPV and HRV are represented using time-domain or frequency-domain indices. Time-domain measures, such as mean, standard deviation and coefficient of variation, quantify the amount of variability in blood pressure or RR-interval measurements over a ≥ 5 min period [16, 106]. In addition to the conventional statistical estimates, blood pressure complexity analysis, which quantifies the irregularity of a signal, has been implemented by measuring the degree of self-similarity or repeated patterns within the signal via entropy-based measures [97, 107]. It has been reported that the physiologic complexity of the blood pressure signal is reduced with aging and in pathological diseases [108]. Similar to time-domain analysis, a minimum of 5 min continuous physiological signal is required for frequency-domain analysis to guarantee sufficient frequency resolution [109]. Frequency analysis of physiological signals reveals the amount of signal power across different frequencies, which represent separate components of the autonomic nervous system, such as the sympathetic or the parasympathetic pathways [16, 106]. The frequency-domain indices can either be expressed in the power unit (i.e., mm Hg^2^ for BPV and ms^2^ for HRV) or the power spectral density unit (i.e., mmHg^2^/Hz for BPV and ms^2^/Hz for HRV).

#### Hemodynamic changes in response to autonomic challenge tests

Autonomic challenge tests augment autonomic responses, leading to more obvious, measurable changes in beat-to-beat hemodynamic measurements. These changes also reflect variations in the vasculature characteristics. These tests are highly sensitive and specific in determining the functional integrity of the autonomic nervous system in both normotensive and hypertensive patients [110].

#### Arterial stiffness

Arterial stiffness, which refers to the structural or mechanical properties of the arterial system, is believed to influence the physiological variability [31]. Sonographic examination of the carotid arteries allows non-invasive assessment of the vasculature with the measurement of arterial wall thickness commonly used to determine the presence of hypertensive complications. Arterial stiffness can also be evaluated through pulse wave velocity (PWV), augmentation index (AIx), arterial compliance or distensibility coefficient determined through tonometry performed on a peripheral artery, usually the radial, femoral or carotid arteries. Commercially available, validated devices are able to calculate the relevant indices automatically using preset algorithms, providing us with a detailed picture of the arterial stiffness.

### Quality assessment

The Strengthening the Reporting of Observational Studies in Epidemiology (STROBE) guidelines [111] were referenced to assess the risk of bias. Seventeen out of 22 STROBE checklist items were reported to identify the potential sources of bias related to the scope and objectives of this systematic review. The checklist comprised of six main components: abstract, introduction, methods, results, discussions and other information. Two reviewers resolved the discrepancies through discussion.

## Data Availability

All relevant data are contained within the article.

## Abbreviations

Abs: Absolute unit
AIx: Augmentation index
ANS: Autonomic nervous system
ARV: Average real variability
ba-PWV: Brachial–ankle pulse wave velocity
BPV: Blood pressure variability
BPV (complexity): Complexity of systolic/diastolic blood pressure
BRS: Baroreflex sensitivity
cf-PWV: Pulse wave velocity between carotid and femoral arteries
cIMT: Carotid intima–media thickness
CO: Cardiac output
CV: Coefficient of variation
DBP: Diastolic blood pressure
DC: Distensibility coefficient
ESRD: End-stage renal disease
HF: Spectral power at high-frequency band
HR: Heart rate
HRV: Heart rate variability
HUT: Head-up tilt
IMT: Intima–media thickness
LF: Spectral power at low-frequency band
MAP: Mean arterial pressure
MSNA: Muscle sympathetic nervous activity
PP: Pulse pressure
PWV: Pulse wave velocity
Rel: Relative unit
RRI: RR-intervals
RSD: Residual standard deviation
SBP: Systolic blood pressure
SCWT: Stroop Color and Word Test
SD: Standard deviation
SV: Stroke volume
SVR: Systemic venous resistance
SVRI: Systemic venous resistance index
TAC: Total arterial compliance (SV/PP)
TP: Total power
TPR: Total peripheral resistance
TR: Time-rate (first derivative of the BP values against time)
VIM: Variation independent of mean
VM: Valsalva maneuver
